# One-Year Prospective Study of Plasma Biomarkers From CNS in Patients With Mild Traumatic Brain Injury

**DOI:** 10.3389/fneur.2021.643743

**Published:** 2021-04-21

**Authors:** Gerard Janez Brett Clarke, Toril Skandsen, Henrik Zetterberg, Cathrine Elisabeth Einarsen, Casper Feyling, Turid Follestad, Anne Vik, Kaj Blennow, Asta Kristine Håberg

**Affiliations:** ^1^Department of Radiology and Nuclear Medicine, St. Olavs Hospital, Trondheim University Hospital, Trondheim, Norway; ^2^Department of Neuromedicine and Movement Science, Faculty of Medicine and Health Sciences, Norwegian University of Science and Technology, NTNU, Trondheim, Norway; ^3^Department of Physical Medicine and Rehabilitation, St. Olavs Hospital, Trondheim University Hospital, Trondheim, Norway; ^4^Department of Psychiatry and Neurochemistry, Institute of Neuroscience and Physiology, The Sahlgrenska Academy at the University of Gothenburg, Mölndal, Sweden; ^5^Clinical Neurochemistry Laboratory, Sahlgrenska University Hospital, Mölndal, Sweden; ^6^Department of Neurodegenerative Disease, University College London Queen Square Institute of Neurology, London, United Kingdom; ^7^UK Dementia Research Institute at University College London, London, United Kingdom; ^8^Department of Public Health and Nursing, Norwegian University of Science and Technology (NTNU), Trondheim, Norway; ^9^Department of Neurosurgery, St. Olavs Hospital, Trondheim University Hospital, Trondheim, Norway

**Keywords:** concussion, axonal injury, astrocytic injury, mixed-mechanism mild TBI, soft tissue injury

## Abstract

**Objective:** To investigate the longitudinal evolution of three blood biomarkers: neurofilament light (NFL), glial fibrillary acidic protein (GFAP) and tau, in out-patients and hospitalized patients with mild traumatic brain injury (mTBI) compared to controls, along with their associations—in patients—with clinical injury characteristics and demographic variables, and ability to discriminate patients with mTBI from controls.

**Methods:** A longitudinal observation study including 207 patients with mTBI, 84 age and sex-matched community controls (CCs) and 52 trauma controls (TCs). Blood samples were collected at 5 timepoints: acute (<24 h), 72 h (24–72 h post-injury), 2 weeks, 3 and 12 months. Injury-related, clinical and demographic variables were obtained at inclusion and brain MRI within 72 h.

**Results:** Plasma GFAP and tau were most elevated acutely and NFL at 2 weeks and 3 months. The group of patients with mTBI and concurrent other somatic injuries (mTBI+) had the highest elevation in all biomarkers across time points, and were more likely to be victims of traffic accidents and violence. All biomarkers were positively associated with traumatic intracranial findings on MRI obtained within 72 h. Glial fibrillary acidic protein and NFL levels were associated with Glasgow Coma Scale (GCS) score and presence of other somatic injuries. Acute GFAP concentrations showed the highest discriminability between patients and controls with an Area Under the Curve (AUC) of 0.92. Acute tau and 2-week NFL concentrations showed moderate discriminability (AUC = 0.70 and AUC = 0.75, respectively). Tau showed high discriminability between mTBI+ and TCs (AUC = 0.80).

**Conclusions:** The association of plasma NFL with traumatic intracranial MRI findings, together with its later peak, could reflect ongoing secondary injury or repair mechanisms, allowing for a protracted diagnostic time window. Patients experiencing both mTBI and other injuries appear to be a subgroup with greater neural injury, differing from both the mTBI without other injuries and from both control groups. Acute GFAP concentrations showed the highest discriminability between patients and controls, were highly associated with intracranial traumatic injury, and showed the largest elevations compared to controls at the acute timepoint, suggesting it to be the most clinically useful plasma biomarker of primary CNS injury in mTBI.

## Introduction

Finding minimally invasive, cost-effective, CNS-specific, objective biomarkers capable of assessing mild traumatic brain injury (mTBI) pathology would greatly improve the clinical care of this group of patients that make up over 90% (1) of the 69 million annual TBI cases (2). The ideal biomarker should be CNS-specific, released as a consequence of injury, be correlated with severity of injury and provide information on the phase in the evolution of the TBI injury (3). Today, Ubiquitin C-terminal hydrolase L1 (UCH-L1) and glial fibrillary acidic protein (GFAP) are approved by the FDA in the US to determine the presence of intracranial injury in mTBI during the acute phase (4, 5), and S100B is approved in Scandinavia for the triage of patients with mTBI to CT scanning during the first 24 h after injury (6). Blood biomarkers with such a short time window are suboptimal for mTBI as many patients present later to their general practitioner or the emergency room. Moreover, S100B is known to be released also from orthopedic/soft tissue injuries (7), and recent evidence points to GFAP being less CNS-specific than previously thought (7).

In the present study, we investigated the temporal evolution of three biomarkers in peripheral blood: glial fibrillary acidic protein (GFAP), neurofilament light (NFL) and tau, from the acute phase across a period of 12 months in patients with mTBI, trauma controls (TCs) and community controls (CCs). The patients with mTBI were further stratified into two groups: those with an isolated mTBI without other injuries (mTBI–) and those sustaining other somatic injuries (e.g., dislocations, fractures, soft tissue injuries in need of treatment) (mTBI+). A more granular investigation of the long-term evolution of biomarkers is relevant, as studies have shown significantly elevated concentrations of inflammatory markers in mTBI for up to 1 year following injury (8), and that collecting blood biomarkers prospectively increases sensitivity and specificity (3).

Glial fibrillary acidic protein is a TBI blood-based biomarker reflecting astrocytic damage (9, 10). It is detectable in blood 1 h following sports-related and mixed-mechanism mTBI, and has been shown to remain significantly elevated for up to 2 weeks (11–13). Glial fibrillary acidic protein discriminates patients with mTBI from controls and patients with traumatic intracranial findings from those without (4, 7, 9, 12–19). Further, recent evidence indicates that GFAP exhibits graded elevation across three injury types, with non-concussive body trauma showing the lowest GFAP concentrations, non-concussive head trauma in the middle, to those with concussion demonstrating the highest GFAP concentrations (20). This points to the importance of investigating the effect of other somatic injuries on biomarker concentrations.

NFL is considered to reflect secondary axonal injury (21). Prior studies have demonstrated its ability to discriminate patients with mild TBI from controls and also patients with traumatic intracranial findings from those without (9, 14, 16, 19). In sports concussion, NFL has been shown to increase over the course of 6–13 days post-injury and remain elevated for up to 3 months (13, 22, 23). A recent published paper indicated that NFL can remain significantly elevated compared to controls for up to 5 years post-injury (14). NFL thus represents a biomarker of the hallmark secondary axonal injury in TBI and/or protracted CNS reparative processes, and could thus be a potential biomarker with a protracted time window. Its long-term time course in mTBI and its associations with clinical (e.g., intracranial findings and other bodily injuries) and demographic variables (e.g., age and sex) are a nascent area of research.

Tau is an important biomarker in severe or repeated head injury (24–26), but studies investigating the discriminability of peripherally-measured tau in patients with mTBI have yielded mixed results (13, 23, 27–29). Furthermore, the long-term time course of tau in mTBI remains under-investigated. One study reported elevated tau in the chronic phase after severe TBI (30), while another showed elevated tau in concussed Australian football players compared to controls at 13 days post-injury (13). A previous study showed no difference in tau concentrations between individuals with mTBI and those with other somatic injuries (31), however a comparison between patients with mTBI, TCs, and CCs has not yet been performed.

The aims of this study were to: (1) provide novel insight into the temporal evolution of plasma-derived GFAP, NFL, and tau in mixed-mechanism mTBI, from the acute phase to 12 months post-injury compared to controls; (2) provide insights into the biomarkers' associations with demographic and typical mTBI injury characteristics, such as intracranial findings on brain MRI within 72 h; (3) further investigate the impact of other somatic injuries on the biomarker concentrations in the mTBI–, mTBI+, CC, and TC groups; (4) assess the ability of each biomarker to classify patients with mTBI vs. combined control group, and between the mTBI+ vs. TC group.

## Materials and Methods

### Participants and Recruitment

The Trondheim mTBI study is a prospective, observational cohort study with follow up for 12 months in patients with mTBI and matched CCs and TCs between 16 and 60 years of age. A total of ~200 patients, ~80 CCs, and ~80 TCs were to be recruited into this arm of the study which included brain MRI (not performed on the TCs), blood sampling, clinical and neuropsychological follow up across 12 months.

Participants were included from April 1st 2014 and the last 12-month follow up was performed November 30th 2018. Patients with mTBI were recruited from two emergency departments (EDs) to reduce bias: St. Olavs hospital (Trondheim University Hospital), a regional level 1 trauma center in Trondheim, Norway, and Trondheim Municipal Emergency clinic, a general practitioner-run, 24-h/7-day out-patient clinic. All patients are triaged by first responders in person or over the phone and assigned to be evaluated further either at hospital or general partitioner run ED. The EDs are located in the same building, use a common CT scanner and the same radiological service, and the healthcare personnel have received the same training and follow the same national and regional guidelines. Most patients with mTBI (≈80%) are triaged to evaluation in the general practitioner-run ED. In the case of traumatic intracranial findings on CT, or clinical conditions, including other injuries in need of specialist care, the patient is referred to the hospital. Approximately 20% of patients with mTBI are admitted directly to the hospital's ED (32, 33).

In this study, inclusion criteria were having sustained an mTBIm, in line with the position statement (34), and classified as mild according to World Health Organization criteria (35), i.e. Glasgow Coma Scale (GCS) score of 13–15, no loss of consciousness (LOC) or <30 min and no post-traumatic amnesia (PTA) or <24 h. Exclusion criteria were: (1) non-fluency in the Norwegian language, (2) pre-existing neurological, psychiatric, somatic, or substance use disorder; determined to be severe enough to interfere with follow-up and outcome assessment, (3) a prior history of a complicated mild (i.e., having trauma-related intracranial findings on CT or MRI), moderate or severe TBI, (4) other major trauma that could interfere with follow-up or outcome assessment, or (5) presentation > 48 h after the initial trauma. See references (32) and (33) for more details on the representativeness of the cohort.

### Clinical Information

Clinical information was obtained from patient interviews and medical records. Clinical variables were assessed using standard protocols. LOC was rated as present only if observed. Duration of PTA was recorded as time after injury for which the patient had no continuous memory (<1 h, or 1–24 h). Glasgow Coma Scale score was assessed in the ED or inferred from records (36). Presence of injuries to parts of the body other than the head (e.g., dislocations, fractures, and soft tissue injuries in need of treatment) was recorded based on self-report and ED/hospital records. Skin abrasions and contusions were not included in this rating. The other somatic injuries were divided into those pertaining to the head, and to those pertaining to the body below the neck. Injuries below the neck that could be present in both mTBI+ and TC groups are: fractures, musculoskeletal/ligament/soft tissue injuries (sprains, torn or ruptured ligaments, capsular injuries, dislocations), internal organ injury (tear, rupture, contusion of internal organs such as spleen, lung), sutured or otherwise treated wounds and unspecified (details on the specifics are lacking). Since a neck/face/head injury was an exclusion criterion for the TC group, presence of musculoskeletal/ligament/soft tissue injuries and wounds in need of suturing to this region are only reported for the mTBI+ group. Note that many patients had several types of injuries, and only the most serious is included in the injury characterizes overview.

### Neuroimaging

Acute phase non-contrast head CT was performed as part of the clinical assessment (6) and head MRI obtained within 72 h in participants with blood drawn. All subjects included underwent a standardized brain MRI scan within this timeframe (33). Since MRI has been shown to be more sensitive to intracranial traumatic findings (33), the results from the clinical MRI readings were used here. All MRI scans were acquired with the same protocol on the same 3.0 Tesla Siemens Skyra MRI scanner with a 32-channel head coil. The protocol included 3D volumes with T1-weighted (Magnetization Prepared Rapid Acquisition Gradient Echo), T2-weighted, Fluid-attenuated inversion recovery, and susceptibility-weighted scans. The clinical scans were read by neuroradiologists according to standard criteria, and the inter-rater reliability was moderate to good (33). Traumatic axonal injury (TAI) was diagnosed and graded as described previously (37). More detailed patient MRI results and their development over time are presented in Einarsen et al. (33).

### Blood Samples

Time of blood sampling was measured as time from injury. Patients with mTBI had their blood drawn either acutely (within 24 h post-injury), or at 72 h (between 24 and 72 h post-injury), then at 2 weeks (±3 days), 3 months (±2 weeks), and 12 months (±1 month). For CCs, blood was sampled at inclusion, corresponding to acute in patients, and after 3 and 12 months. The TCs had their blood sampled acutely, at 72 h, 2 weeks, and 3 months. Plasma samples used in the current study were obtained with EDTA gel tubes immediately put on ice and centrifuged for 10 min at at 4°C on 2,000 g within 30 min of acquisition. The aliquoted plasma samples were stored at −80°C. Plasma concentrations of GFAP, NFL and tau were analyzed using the Human Neurology 4-Plex A assay (N4PA) on an HD-1 Single molecule array (Simoa) instrument according to instructions from the manufacturer (Quanterix, Lexington, MA).

### Statistical Analysis

Frequencies and percentages of demographic and clinical variables for the total number of participants with data available at minimum one timepoint were calculated. Patients with mTBI were split into a group with presence of other somatic injuries (mTBI+) and a group without presence of other somatic injuries (mTBI–). *T*-tests were used to compare the difference in age between mTBI+ and mTBI– groups. Chi square or Fisher exact-tests were used to compare the frequency of different injury mechanisms, GCS scores, LOC, PTA, intoxication and presence of any traumatic intracranial findings on brain MRI, along with the distribution of sex, between the mTBI– and mTBI+ groups. The Chi Square-test operates by comparing the actual cell values to an expected cell value frequency, i.e., the value expected within a cell if all proportions were equal. An assumption of this test is that expected frequencies do not fall below 5 in at least 80% of cells, when this happened with our data, a Fisher exact-test was used instead. The low number of traumatic intracranial findings and co-occurrence of more than one finding necessitated merging findings into a positive MRI category for all analyses. Likewise, the Fisher exact-test was used to compare the frequency of injury mechanisms between the mTBI– and TC groups, and between mTBI+ and TC groups. If the overall Fisher exact-test was significant, *post-hoc*-tests were performed to determine which pairwise comparisons differed significantly.

All biomarker concentrations were log-transformed due to non-normally distributed data. Descriptive statistics (mean, standard deviation, median, interquartile range, and range) of non-log transformed biomarker values were calculated. To assess the viability of merging the CC and TC groups into a combined control group for initial mixed model and ROC analyses, Mann-Whitney *U*-tests were used to compare the three biomarker levels at acute and 3-month timepoints. This test was chosen instead of the *t*-test due to the low sample size of the TC group, which led to the assumption of normality being violated.

Mixed model analyses were conducted on the mTBI group and combined control group with time, group, and a time-by-group interaction as fixed effects, and a subject-specific random intercept to account for within-subject correlations. This analysis is used to assess the time course of multiple groups, allowing comparisons both across timepoints within a certain group, and group comparisons within a given timepoint. Group differences at each timepoint and within-group changes for the mTBI group between successive timepoints were assessed using *post-hoc* contrast tests for the estimated model parameters. Furthermore, Pearson's correlations between early-phase GFAP (acute and 72 h), subacute NFL (2 weeks and 3 months) and acute tau were computed. As acute and 72-h timepoints were sampled independently, a correlation between acute tau and 72-h GFAP was not possible.

Best-subset regression analyses were performed to determine the best combination of clinical (GCS score, PTA duration, LOC, presence of traumatic intracranial MRI findings, somatic injuries) and demographic (sex and age) variables that predict biomarker levels in patients with mTBI. This analysis involves testing all possible models of all combinations of possible predictors input into the model, using a statistical algorithm to determine which model fits the data best. Best model fit was determined based on the Akaike Information Criterion (AIC). The analyses were performed at acute and 72-h timepoints for GFAP, the acute timepoint for tau and at 2 weeks and 3 months for NFL. These timepoints were selected for subsequent analysis as they showed significant group differences in the previous mixed model analysis. The biomarker values were first standardized at each timepoint, then best-subset regression analyses were performed using the standardized biomarkers as outcome variables. Regression coefficients based on unstandardized biomarker values were also calculated. Empirical group means and standard deviations in biomarker levels of patients with mTBI for each categorical predictor used in the best-subset regression analysis were calculated. Additional best-subset regression analyses were performed on a subset of patients with mTBI without intracranial findings on MRI (those with uncomplicated mild TBI).

To assess whether somatic injuries affected blood biomarker levels, we first performed the linear mixed model analysis as described above, but confined to the mTBI– group compared to CC group. We subsequently examined the effect of presence of somatic injuries on the level of GFAP, NFL and tau using one-way ANOVAs to compare biomarker concentrations between mTBI+, mTBI–, CC, and TC groups. Previously selected timepoints were used. If the omnibus test was significant, Tukey's HSD was performed to determine the significant pairwise differences. Since blood was not drawn from CCs at 72 h or 2 weeks, these comparisons were performed between mTBI–, mTBI+, and TCs only. Somatic injuries were further delineated by comparing injury type below the neck between mTBI+ and TCs using a Fisher exact-test.

Receiver-operating characteristic (ROC) curve analyses were conducted to assess the ability of each biomarker to correctly categorize patients with mTBI vs. combined controls and mTBI+ vs. trauma controls. Thresholds of the biomarkers at the previously defined selected timepoints were calculated using Youden's J statistic. A best-subset logistic regression was performed to determine the best combination of biomarkers to discriminate mTBI from combined controls and mTBI+ from TCs. Acute and 72-h timepoints were combined for GFAP and tau to increase statistical power. Therefore, GFAP within 72 h, Tau within 72 h, NFL at 2 weeks and age were included as possible predictors. ROC curve analyses were conducted on the best model. The best-subset regression and ROC analysis described was also conducted using a subset of patients with uncomplicated mTBI (those without intracranial traumatic findings on MRI) compared to combined controls.

Significance tests were two-sided with significance set to *p* < 0.05. *Post-hoc* contrast analyses of the mixed models were Bonferroni corrected. Comparing patients with mTBI to the combined control group, significance level of group differences at each timepoint was 0.05/5 = 0.01. Significance level of within-group changes for patients with mTBI between successive time points was 0.05/4 = 0.0125. The significance level of group comparisons between the mTBI– group and community controls was 0.05/3 = 0.017. Each Fisher-Freeman-Holt exact-test was Bonferroni corrected.

All statistical analyses were performed using R version 3.2.2 (38).

## Results

**Figure 1:**
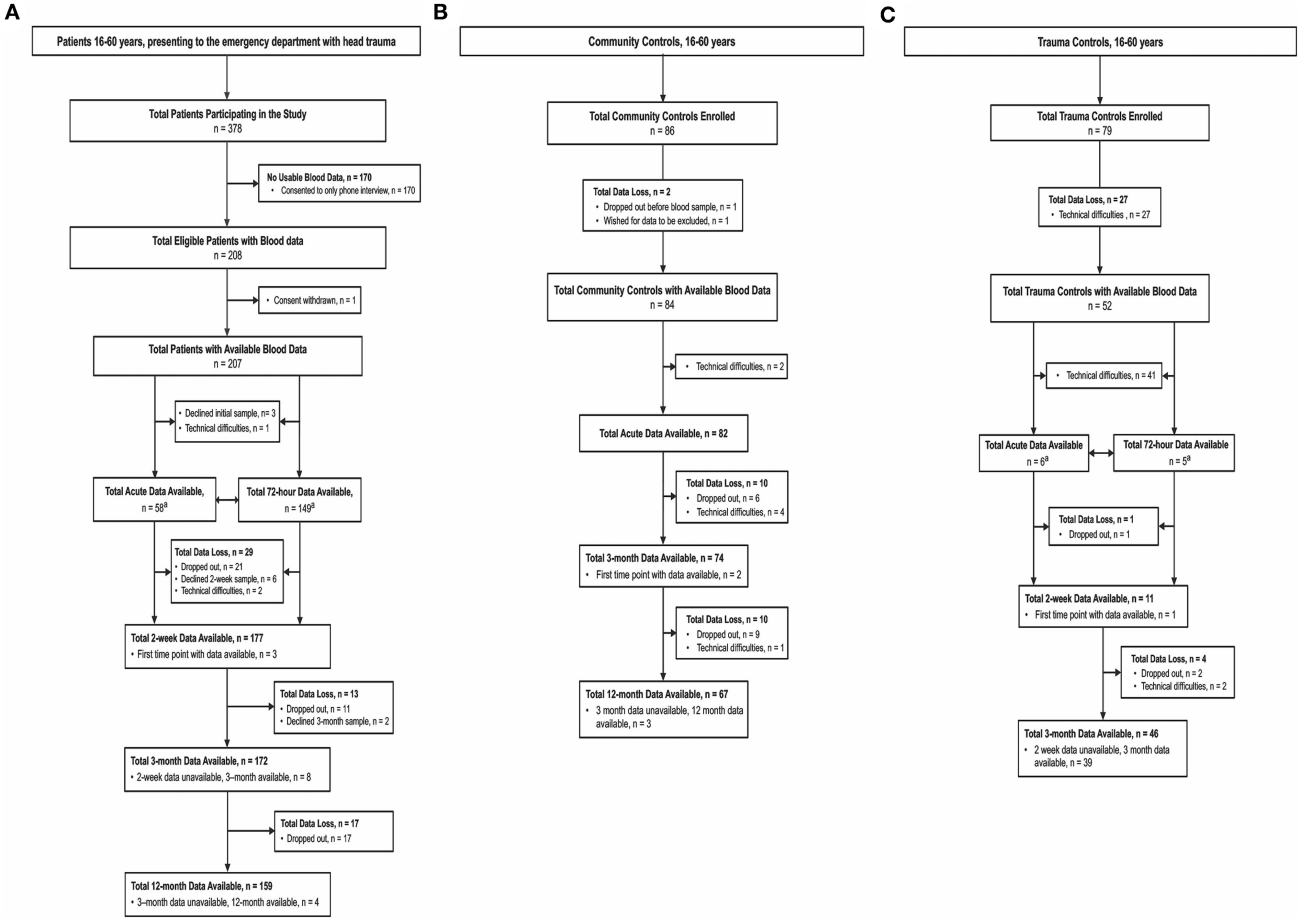
Enrolment and follow-up of **(A)** patients with mTBI, **(B)** Community Controls, and **(C)** Trauma Controls. mTBI, mild traumatic brain injury; CT, computed tomography. ^a^Acute and 72-h blood data are shown next to each other as these time points are largely independent.

The flow charts in Figure 1 summarize the group numbers and reasons for drop out. Two hundred and seven had usable blood data at one or more timepoints. By 12 months, 159 patients with mTBI remained in the study, giving a retention rate of 78%. Of the 86 CCs enrolled, 84 had usable blood data at one or more timepoints. There were 82 CCs with acute data, and 67 at 12 months, giving an 82% retention rate. Of the 79 TCs enrolled, 52 had usable blood data. There were 11 TCs with data at early timepoints (acute, 72 h) and 11 at 2 weeks, but 46 with data available at 3 months.

Table 1 provides a detailed summary of the demographic and injury-related characteristics of the different groups. The majority of patients with mTBI were male (63.3%). The majority had less severe injuries, with GCS scores of 15 in 76.0%. LOC was observed in 52.7% and most had PTA < 1 h (69.1%). Intracranial traumatic findings MRI at 72 h were present in only 11.1% and most (63.3%) did not experience concurrent somatic injuries.

**Table 1:** Characteristics of total number of participants included in the study.

	**mTBI patients**	**mTBI+ patients**	**mTBI– patients**	**Combined controls**	**Community controls**	**Trauma controls**
	***N* = 207**	***N* = 76**	***N* = 131**	***N* = 136**	***N* = 84**	***N* = 52**
**Gender (%)**						
Males	131 (63.3)	49 (64.5)	83 (63.4)	75 (55.1)	48 (57.1)	27 (51.9)
Females	76 (36.7)	27 (35.5)	48 (36.6)	61 (44.9)	36 (42.9)	25 (48.1)
**Age at inclusion**						
Mean age (SD)	32.4 (13.2)	33.2 (14.7)	31.9 (12.4)	32.9 (12.6)	33.2 (13.0)	32.4 (12.2)
Age range	16–60	16–60	16–59	16–60	16–59	17–60
**GCS (%)**						
13	5 (2.4)	1 (1.3)	4 (3.1)			
14	33 (16)	12 (15.8)	21 (16.0)			
15	158 (76)	58 (76.3)	100 (76.3)			
Unknown	11 (5.3)	5 (6.6)	6 (4.6)			
**LOC (%)**						
No LOC observed	109 (52.7)	40 (52.6)	69 (52.7)			
LOC observed	98 (47.3)	36 (47.4)	62 (47.3)			
**PTA (%)**						
PTA <1 h	143 (69.1)	55 (72.4)	88 (67.2)			
PTA between 1 and 24 h	64 (30.9)	21 (27.6)	43 (32.8)			
**Injury mechanism mTBI (%)**						
Fall	79 (38.1)	22 (29.0)	57 (43.5)			19 (36.5)
Traffic accident	57 (27.5)	29 (38.2)	28 (21.4)			2 (3.9)
Sports accident	26 (12.6)	7 (9.2)	19 (14.5)			23 (44.2)
Violence	31 (15.0)	16 (21.1)	15 (11.4)			1 (1.9)
Hit object and other	14 (6.8)	2 (2.6)	12 (9.2)			3 (5.8)
**Intoxication at time of injury (%)**						
Yes	91 (43.8)	32 (42.1)	59 (45.0)			
No	117 (56.2)	44 (57.9)	72 (55.0)			
**Intracranial finding on MRI at 72 h (%)**						
TAI only	6 (2.9)	4 (5.3)	2 (1.5)			
Contusion only	3 (1.4)	1 (1.3)	2 (1.5)			
Intracranial haematoma only	3 (1.4)	1 (1.3)	2 (1.5)			
Both TAI and contusion	5 (2.4)	2 (2.6)	3 (2.3)			
Both contusion and haematoma	6 (2.9)	1 (1.3)	5 (3.9)			
No visible traumatic findings	184 (89)	67 (88.2)	117 (89.3)			
**Somatic injuries (%)**^‡^						
No	131 (63.3)	0 (0)	131 (100)			
Yes	76 (36.7)	76 (100)	0 (0)			52 (100)
**Types of injuries**						
**Non-head-face-neck injury**						
Fractures		6 (8.0)				31 (59.6)
Muscle/ligaments/soft tissue		13 (17.1)				19 (36.6)
Internal		3 (3.9)				0 (0)
Wound sutured		5 (6.6)				2 (3.8)
Unspecified		15 (19.7)				0 (0)
**Head-face-neck injury**						
Fractures		26 (34.2)				
Muscle/ligaments/soft tissue		3 (3.9)				
Wound sutured		5 (6.6)				

*mTBI, mild traumatic brain injury; mTBI+, patients with mTBI and other somatic injuries; mTBI–, patients with mTBI without other somatic injuries; TC, Trauma controls; GCS, Glasgow coma score; LOC, Loss of consciousness; PTA, Post-traumatic amnesia; MRI, Magnetic resonance imaging*.

‡ *Somatic injuries refer to the presence of additional injuries to parts of the body in need of treatment in addition to the mTBI. In cases of multiple injuries, the most serious was recoded here. Note that injuries to head, face and neck were exclusion criteria in the TC group*.

Within the mTBI group, patients without other somatic injuries (mTBI–) and with other somatic injuries (mTBI+) had a similar distribution of age [*t*_(205)_ = −0.67, *p* = 0.51] and sex [$\chi_{\left(1\right)}^{2}$ = 0.03, *p* = 0.88], along with similar frequencies of GCS scores [$\chi_{\left(2\right)}^{2}$ = 0.59, *p* = 0.74], observed LOC [$\chi_{\left(1\right)}^{2}$ = 0.000, *p* = 1.0], duration of PTA [$\chi_{\left(1\right)}^{2}$ = 0.61, *p* = 0.53], presence of intoxication [$\chi_{\left(1\right)}^{2}$ = 0.17, *p* = 0.77], and presence of traumatic intracranial findings on MRI [$\chi_{\left(1\right)}^{2}$ = 0.06, *p* = 0.82].

Injury mechanisms varied significantly between the mTBI+ and mTBI– groups [$\chi_{\left(4\right)}^{2}$ = 14.33, *p* = 0.006]. The mTBI+ group was significantly more frequently involved in traffic accidents (*p* = 0.009) than the mTBI– group and there was a trend toward the mTBI– group experiencing fewer falls (*p* = 0.037). Injury mechanisms in the TC group differed significantly from those in both the mTBI– group [$\chi_{\left(4\right)}^{2}$ = 26.05, *p* = <0.0001] and the mTBI+ group [$\chi_{\left(4\right)}^{2}$ = 48.64, *p* = <0.0001]. Individuals in the TC group were significantly more often involved in sport-related accidents (*p* < 0.0001) and less frequently in traffic accidents (*p* = 0.004) compared to the mTBI– group. Compared to the mTBI+ group, the TC group was also significantly more frequently involved in sporting accidents (*p* < 0.0001) and less often in traffic accidents (*p* < 0.0001) or violence (*p* = 0.002).

Head-face-neck injuries were very common in the mTBI+ group, but not present in the TC group as this was an exclusion criterion. Type of other bodily injuries varied significantly between TCs and patients with mTBI (Fisher's exact-test, *p* < 0.0001). TCs had significantly more fractures than the mTBI+ group (*p* < 0.0001), while the mTBI+ group had significantly more unspecified injuries (*p* < 0.0001).

### Longitudinal Evolution of Blood Biomarkers From the Acute Phase to 12 Months Post-Injury

Mann-Whitney *U*-tests (Supplementary Table 1) revealed no significant differences in any biomarker concentrations between CC and TC groups at neither acute nor 3-month timepoints. Therefore, the groups were merged into one control group for subsequent mixed models and initial ROC analyses, but not the analyses pertaining specifically to somatic injury.

**Figure 2:**
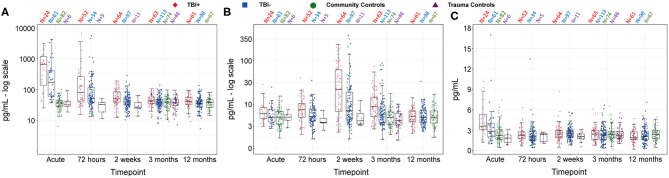
Concentrations of GFAP **(A)**, NFL **(B)**, and tau **(C)** over time, for mTBI+, mTBI–, trauma controls and community controls. Data are presented as box plots with median as the midline, box borders representing the 25th and 75th percentile and whiskers calculated as the 25th and 75th percentile + 1.5 * interquartile range. Points above and below the whiskers represent outliers. Individual data points are presented within the box-plots. GFAP and NFL are presented on a log-transformed scale, while tau retains its original scale, for visualization purposes. GFAP, Glial fibrillary acidic protein; NFL, Neurofilament light; mTBI+, patients with mTBI and presence of other somatic injuries; mTBI–, patients with mTBI and no presence of other somatic injuries. *N*, number of participants in each group with blood at each timepoint.

Figure 2 presents the longitudinal evolution of the biomarkers. Table 2 presents *post-hoc* contrasts between patients with mTBI and the combined control group based on a linear mixed model, and Table 3 shows *post-hoc* contrasts of patient biomarker concentrations over time (main effects of the linear mixed model are presented in Supplementary Table 2).

**Table 2:** Group comparisons of biomarker concentrations between patients with mTBI and controls at each time point, calculated using *post-hoc* contrast analyses based on the linear mixed model results.

	**Acute**	**72 h**	**2 weeks**	**3 months**	**12 months**
	**Estimate^‡^ (95% CI) *p*-value**	**Estimate^‡^ (95% CI) *p*-value**	**Estimate^‡^ (95% CI) *p*-value**	**Estimate^‡^ (95% CI) *p*-value**	**Estimate^‡^ (95% CI) *p*-value**
	**Mean % elevation**	**Mean % elevation**	**Mean % elevation**	**Mean % elevation**	**Mean % elevation**
GFAP	0.84 (0.75 to 0.94) ***p*** **< 0.0001**	0.46 (0.21 to 0.71) ***p*** **= 0.0004**	0.20 (0.02 to 0.38) *p* = 0.030	0.05 (−0.03 to 0.12) *p* = 0.232	0.02 (−0.06 to 0.11) *p* = 0.599
	56.5%	34.7%	12.7%	2.3%	1.8%
NFL	0.06 (−0.03 to 0.15) *p* = 0.190	0.14 (−0.09 to 0.37) *p* = 0.238	0.43 (0.26 to 0.59) ***p*** **< 0.0001**	0.21 (0.14 to 0.28) ***p*** **< 0.0001**	0.01 (−0.07 to 0.10) *p* = 0.735
	11.9%	27.1%	60.9%	30.8%	1.2%
Tau	0.19 (0.12 to 0.25) ***p*** **< 0.0001**	0.01 (−0.16 to 0.19) *p* = 0.884	0.12 (0.00 to 0.24) *p* = 0.055	−0.01 (−0.06 to 0.04) *p* = 0.682	−0.05 (−0.11 to 0.01) *p* = 0.087
	58.6%	16.5%	40.4%	−3.5%	−14.4%

‡ *Estimate refers to mean group differences as estimated by the mixed model; 95% CI is the 95% confidence interval of the estimated group difference. All values are log transformed. Mean % elevation is the percentage elevation in the mean log-transformed biomarker values of patients with mTBI compared to controls. Significant differences are bolded. Bonferroni-adjusted alpha level = 0.01*.

*mTBI, mild traumatic brain injury; GFAP, Glial fibrillary acidic protein; NFL, Neurofilament light*.

**Table 3:** Differences in biomarker concentrations between time points in patients with mTBI, calculated using *post-hoc* contrast analyses based on the linear mixed model results.

**Biomarkers**	**Acute−72 h**	**72 h−2 weeks**	**2 weeks−3 months**	**3–12 months**
	**Estimate^‡^ (95% CI)**	**Estimate^‡^ (95% CI)**	**Estimate^‡^ (95% CI)**	**Estimate^‡^ (95% CI)**
	***p*-value**	***p*-value**	***p*-value**	***p*-value**
GFAP	−0.45 (−0.53 to −0.37)	−0.25 (−0.30 to −0.20)	−0.09 (−0.14 to −0.04)	−0.02 (−0.07 to 0.03)
	***p*** **< 0.0001**	***p*** **< 0.0001**	***p*** **= 0.001**	*p* = 0.405
NFL	0.06 (−0.01 to 0.14)	0.30 (0.26 to 0.35)	−0.23 (−0.27 to −0.19)	−0.17 (−0.21 to −0.12)
	*p* = 0.086	***p*** **< 0.0001**	***p*** **< 0.0001**	***p*** **< 0.0001**
Tau	−0.20 (−0.25 to −0.14)	0.06 (0.03 to 0.10)	−0.04 (−0.07 to −0.01)	−0.05 (−0.08 to −0.01)
	***p*** **< 0.0001**	***p*** **= 0.001**	*p* = 0.024	***p*** **= 0.011**

‡ *Estimate refers to mean timepoint differences as estimated by the mixed model; 95% CI is the 95% confidence interval of the estimated timepoint difference. All values are log transformed. Significant differences are bolded. Bonferroni-adjusted alpha level = 0.0125*.

*mTBI, mild traumatic brain injury; GFAP, Glial fibrillary acidic protein; NFL, Neurofilament light*.

Glial fibrillary acidic protein concentrations were significantly elevated in patients with mTBI compared to the combined control group at acute and 72-h timepoints (Table 2). Mean log-scale GFAP-values of patients with mTBI were 56.5% higher than controls at the acute timepoint and 34.7% higher at 72 h. Contrasts comparing GFAP levels over time in patients with mTBI revealed large, significant decreases between the acute and 72-h timepoints, the 72-h and 2-week timepoints, and a smaller, but significant decrease between the 2-week and 3-month timepoints (Table 3). There was no significant difference between 3- and 12-month timepoints.

There were large group differences in NFL concentrations at 2-week and 3-month timepoints, and smaller non-significant differences at other timepoints (Table 2). Mean log-scale NFL values of patients with mTBI were 11.9% higher than controls at the acute timepoint, 27.1% higher at 72 h, 60.9% higher than controls at 2 weeks and 30.8% higher at 3 months. There was no significant difference in patient NFL concentrations between acute and 72-h timepoints (Table 3), however patient NFL concentrations significantly increased between 72 h and 2 weeks, significantly decreased between the 2-week and 3-month timepoints, and significantly decreased again between 3 and 12 months.

Plasma tau levels differed significantly between patient and control groups only at the acute timepoint, with a 58.6% elevation of mean tau in patients with mTBI vs. the combined control group (Table 2). Tau levels decreased quickly in the mTBI group, and were significantly lower at 72 h compared to the acute timepoint (Table 3). Tau levels then increased between 72 h and 2 weeks, did not significantly change between 2 weeks and 3 months, and significantly decreased between 3 and 12 months. This shows a large degree of variability in tau levels over time in patients with mTBI.

All correlations between acute GFAP, acute tau and chronic NFL were significant. Acute GFAP was significantly correlated with 2-week NFL (0.89, *p* < 0.0001) and 3-month NFL (0.77, *p* < 0.0001). Seventy-two-hour GFAP was also significantly correlated with 2-week NFL (0.77, *p* < 0.0001) and 3-month NFL (0.72, *p* < 0.0001). Acute tau was significantly correlated with 2-week NFL (0.39, *p* = 0.008), 3-month NFL (0.37, *p* = 0.01) and acute GFAP (0.37, *p* = 0.004).

### The Demographic and Clinical Variables Most Predictive of Biomarker Concentrations in Patients With mTBI at Selected Timepoints

**Figure 3:**
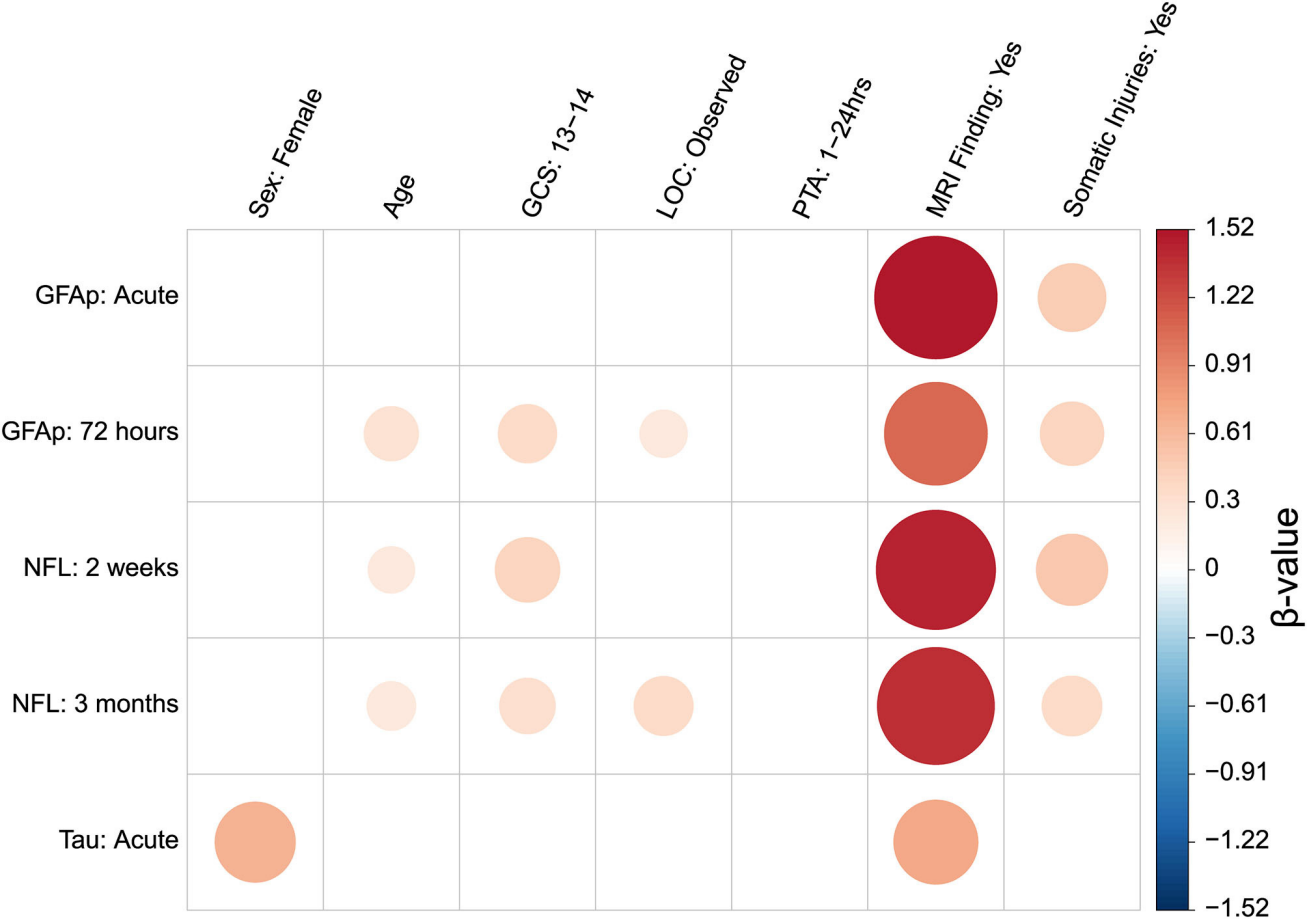
Graphical representation of associations between biomarkers measured at selected timepoints and demographic and clinical variables. Biomarker levels at selected timepoints were input into an all-subsets multiple regression as outcome variables. Demographic and clinical variables were input as possible predictor variables. Associations shown to significantly improve model fit are depicted as colored circles. Best model fit was determined based on the Akaike Information Criterion. The direction of association is represented according to the figure legend color scheme, whereby a red color indicates a positive association and blue indicates a negative association. The size of regression coefficient is represented by both circle size and color gradient. A larger circle indicates a stronger association. Increasingly positive associations are graded to darker red, while increasingly negative associations are graded to darker blue. A blank space indicates that the predictor was not included in the final model. Associations presented by the colored circles are the beta values of the final model. The beta values are regression coefficients from models with standardized biomarker concentrations as outcome. Unstandardized beta and *p*-values are presented in Supplementary Table 4. Means and standard deviations of biomarker levels separated by each categorical variable are presented in Supplementary Table 5. GFAP, Glial fibrillary acidic protein; NFL, Neurofilament light; GCS, Glasgow coma score; LOC, Loss of consciousness; PTA, Post-traumatic Amnesia; MRI, Magnetic resonance imaging. Baseline comparison for Sex was male. Baseline comparison for GCS scores of 13–14 was GCS score of 15. Baseline comparison for LOC observed was “No LOC observed.” Baseline comparison for PTA duration of between 1 and 24 h was PTA of <1 h. Baseline comparison for MRI findings was “No MRI finding.” Baseline comparison Somatic injuries was “No somatic injuries”.

Figure 3 illustrates the associations between the clinical and demographic predictors and the three biomarkers in patients with mTBI at selected timepoints. The unstandardized beta-values and *p*-values are presented in Supplementary Table 5. Supplementary Table 6 presents means and standard deviations of non-log-transformed biomarker concentrations separated by demographic and injury-related variables.

The predictors included in the best-subset model for acute GFAP concentrations were presence of positive traumatic findings on brain MRI and somatic injuries. For GFAP at 72 h, positive MRI findings, GCS score, presence of observed LOC, somatic injuries and age were included in the best model. The models for NFL concentrations at 2 weeks and 3 months included positive MRI findings, somatic injuries, age and GCS score. The 3-month model additionally included LOC. The model for acute tau included positive MRI findings and sex.

When only patients without intracranial findings on MRI (i.e., patients with uncomplicated mTBI) were included in the analyses, the best model to predict acute GFAP concentrations included other somatic injuries only. For GFAP at 72 h, age, LOC, PTA, and somatic injuries were included in the best model. The models for NFL concentrations at 2 weeks and 3 months included somatic injuries, age and LOC. The 3-month model additionally included PTA. The model for acute tau included sex and somatic injuries.

### The Effect of Other Injuries on Plasma Biomarker Levels

#### mTBI– vs. CC

Table 4 shows *post-hoc* contrasts comparing mTBI– patients with CC group at all timepoints (main effects are presented in Supplementary Table 3). The results of the analysis were similar to those of the entire mTBI and combined control group. GFAP was significantly increased in mTBI– compared to CCs in the acute phase and comparable to control levels thereafter (Table 4). NFL was significantly elevated in the mTBI– group at 3 months, but at control levels in the acute phase and after 12 months. Tau levels were significantly elevated in the acute phase.

**Table 4:** Group comparisons of biomarker concentrations between patients with mTBI without somatic injuries (mTBI–) and community controls, at acute, 3 and 12 months, calculated using contrast analyses based on the linear mixed model results.

	**Acute**	**3 months**	**12 months**
	**Estimate^‡^ (95% CI) *p*-value**	**Estimate^‡^ (95% CI) *p*-value**	**Estimate^‡^ (95% CI) *p*-value**
	**Mean % elevation**	**Mean % elevation**	**Mean % elevation**
GFAP	0.73 (0.63 to 0.83), ***p*** **< 0.0001**	0.01 (−0.07 to 0.09), *p* = 0.257	−0.001 (−0.08 to 0.08), *p* = 0.980
	47.6%	1.0%	0.8%
NFL	0.03 (−0.06 to 0.11), *p* = 0.518	0.14 (0.07 to 0.21), ***p*** **= 0.0001**	0.01 (−0.07 to 0.08), *p* = 0.889
	6.8%	19.8%	1.5%
Tau	0.14 (0.05 to 0.23) ***p*** **= 0.002**	−0.03 (−0.10 to 0.04) *p* = 0.374	−0.06 (−0.13 to 0.02) *p* = 0.137
	41.6%	−6.3%	−12.3%

‡ *Estimate refers to mean group differences as estimated by the mixed model; 95% CI is the 95% confidence interval of the estimated group difference. All values are log transformed. Mean % elevation is the percentage elevation in the mean log-transformed biomarker values of patients with mTBI compared to controls. Significant differences are bolded. Bonferroni-adjusted alpha level = 0.017*.

*mTBI, mild traumatic brain injury; GFAP, Glial fibrillary acidic protein; NFL, Neurofilament light*.

#### Comparisons Between mTBI+, TBI–, CC, and TC Groups at Selected Timepoints

All one-way ANOVA main effects comparing the 4 groups were significant (Supplementary Table 4). *Post-hoc* Tukey HSD comparisons are presented in Table 5.

**Table 5:** Results from Tukey HSD comparisons for biomarkers at designated timepoints between patients with mTBI without somatic injuries (mTBI–), patients with mTBI with somatic injuries (mTBI+), trauma controls (TC), and community controls (CC).

**Biomarkers**	**GFAP—acute**	**GFAP–72 h**	**NFL–2 weeks**	**NFL–3 months**	**Tau—acute**
	**Estimate^‡^ (95% CI)**	**Estimate^‡^ (95% CI)**	**Estimate^‡^ (95% CI)**	**Estimate^‡^ (95% CI)**	**Estimate^‡^ (95% CI)**
	***p*-value**	***p*-value**	***p*-value**	***p*-value**	***p*-value**
TC: CC	0.02 (−0.43 to 0.47)			−0.05 (−0.18 to 0.09)	−0.11 (−0.38 to 0.16)
	*p* = 0.99			*p* = 0.83	*p* = 0.70
mTBI–: CC	0.73 (0.51 to 0.95)			0.14 (0.03 to 0.25)	0.13 (0.01 to 0.27)
	***p*** **< 0.0001**			***p =*** **0.008**	***p =*** **0.03**
mTBI+: CC	1.07 (0.82 to 1.31)			0.28 (0.16 to 0.41)	0.25 (0.10 to 0.39)
	***p*** **< 0.0001**			***p*** **< 0.0001**	***p =*** **0.0001**
mTBI–: TC	0.71 (0.24 to 1.18)	0.42 (−0.15 to 0.99)	0.31 (−0.07 to 0.68)	0.18 (0.05 to 0.31)	0.25 (−0.03 to 0.53)
	***p =*** **0.0008**	*p* = 0.20	*p* = 0.13	***p =*** **0.002**	*p =* 0.09
mTBI+: TC	1.05 (0.56 to 1.53)	0.64 (0.06 to 1.22)	0.63 (0.24 to 1.01)	0.33 (0.19 to 0.47)	0.36 (0.07 to 0.65)
	***p*** **< 0.0001**	***p =*** **0.03**	***p =*** **0.0005**	***p*** **< 0.0001**	***p*** **= 0.009**
mTBI+: mTBI–	0.33 (0.05 to 0.62)	0.22 (0.01 to 0.43)	0.32 (0.14 to 0.51)	0.15 (0.03 to 0.26)	0.11 (−0.06 to 0.28)
	***p =*** **0.01**	***p =*** **0.04**	***p =*** **0.0002**	***p =*** **0.008**	*p =* 0.36

‡ *Estimate refers to mean log-transformed group differences as estimated by Tukey's HSD; 95% CI is the 95% confidence interval of the estimated group difference. Significant differences are bolded*.

*mTBI, mild traumatic brain injury; GFAP, Glial fibrillary acidic protein; NFL, Neurofilament light; TC, Trauma controls; CC, Community control*.

At the acute timepoint, GFAP levels were elevated in mTBI+ and mTBI– compared to both control groups, and were significantly higher in mTBI+ compared to the mTBI– group. There was no difference between the two control groups. For the 72-h timepoint, with only blood available from the TC group, the mTBI+ group had increased GFAP levels compared to both the TC and mTBI– groups while the mTBI– group GFAP levels were comparable to the TC group.

For NFL at 2 weeks, the mTBI+ group had higher levels than the TC and mTBI– groups. There was no difference between the TC and mTBI– groups. At the 3-month time point, NFL levels were higher in both mTBI+ and mTBI– groups compared to both CCs and TCs, and higher in mTBI+ compared to mTBI–, while the two control groups were comparable.

For tau in the acute phase, both mTBI+ and mTBI– had higher levels than the CC group, but only mTBI+ (not mTBI–) had higher levels than the TC group. mTBI+ did not differ from mTBI–, nor did TCs differ from CCs.

### Biomarkers' Discriminability Based on ROC Analyses at Selected Timepoints

**Figure 4:**
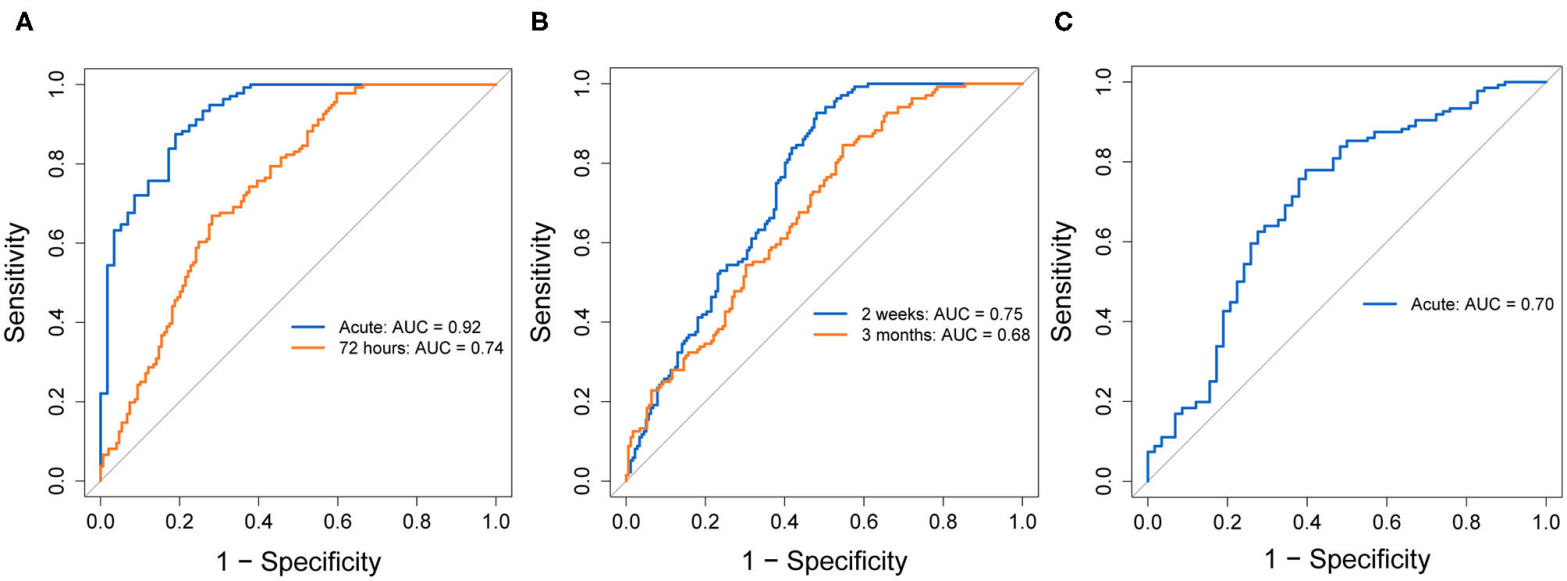
ROC curves indicating diagnostic accuracy of GFAP **(A)**, NFL **(B)**, and tau **(C)**, at selected timepoints, discriminating patients with mTBI from combined controls. ROC curves for each biomarker's evaluated timepoints are overlain on the same plot. AUC values and their 95% confidence intervals are indicated in each plot, for each timepoint. GFAP, Glial fibrillary acidic protein; NFL, Neurofilament light; ROC, Receiver operating characteristic; AUC, Area under the curve; CI, Confidence interval.

**Figure 5:**
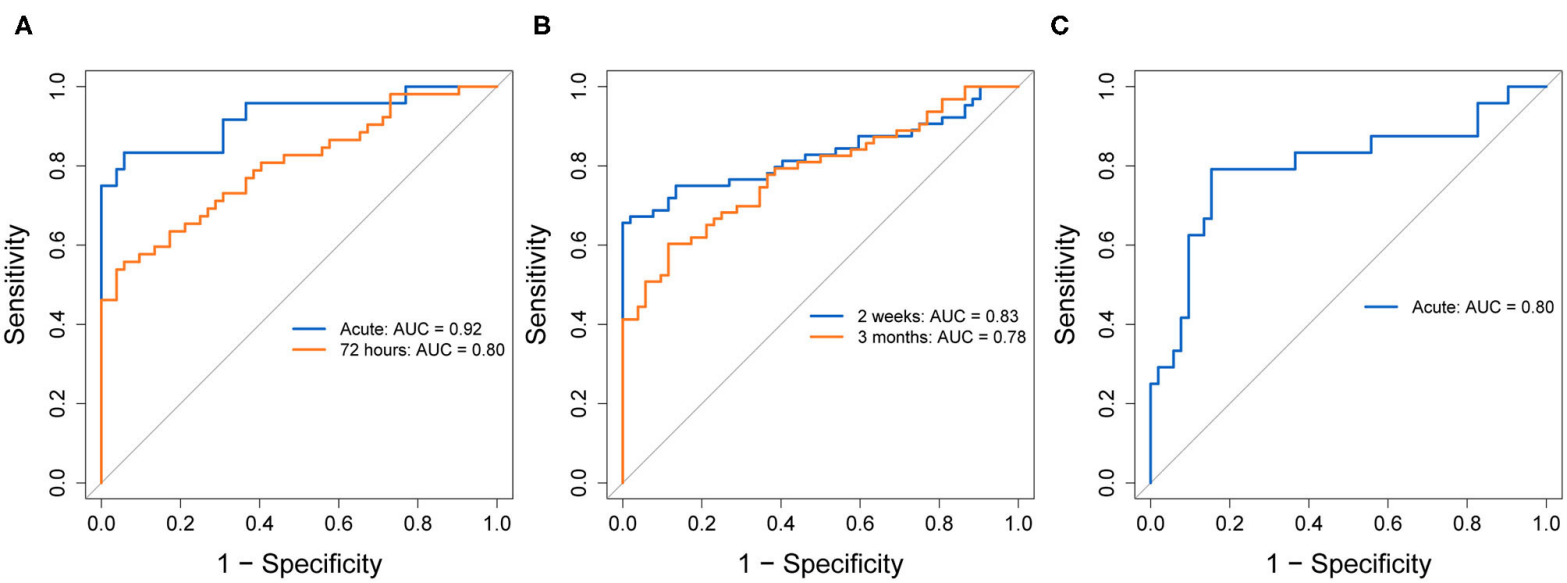
ROC curves indicating diagnostic accuracy of GFAP **(A)**, NFL **(B)**, and tau **(C)**, at selected timepoints, discriminating the mTBI+ group from trauma controls. ROC curves for each biomarker's evaluated timepoints are overlain on the same plot. AUC values and their 95% confidence intervals are indicated in each plot, for each timepoint. GFAP, Glial fibrillary acidic protein; NFL, Neurofilament light; ROC, Receiver operating characteristic; AUC, Area under the curve; CI, Confidence interval.

Figure 4 shows ROC curves comparing patients with mTBI to combined controls for each biomarker at the selected timepoints. Figure 5 shows ROC curves comparing patients with mTBI with other injuries (mTBI+) to TCs. Supplementary Tables 7, 8 provide AUC values, sensitivities, specificities and thresholds for the optimal cut-off based on Youden's J Statistic.

In patients with mTBI vs. combined controls, GFAP at the acute timepoint showed the highest discriminability of all biomarkers, with an AUC of 0.92 and good sensitivity (0.88) and specificity (0.81). At 72 h, GFAP still exhibited good, but lower discriminability (AUC = 0.74), with a lower sensitivity (0.67) and specificity (0.72). Comparing mTBI+ to TCs, acute and 72-h GFAP showed similar AUCs (0.92, 0.80, respectively), with lower sensitivities (0.83, 0.56, respectively) and higher specificities (0.94 for both timepoints).

In patients with mTBI vs. combined controls, NFL showed adequate discriminability at 2 weeks (AUC = 0.75) and poorer discriminability at 3 months (AUC = 0.68). At 2 weeks, sensitivity was very high (0.93), however specificity was low (0.52). At 3 months, NFL sensitivity was moderate (0.60) and specificity was high (0.88). Comparing mTBI+ to TCs, NFL showed higher AUC at both timepoints (0.83, 0.78, respectively), however the sensitivity vs. specificity profile was reversed. Specificity at 2 weeks was perfect (1.00), and very good at 3 months (0.88), while sensitivity was decreased compared to the previous model at both timepoints (0.66, 0.60, respectively).

In patients with mTBI vs. combined controls, tau's AUC at the acute timepoint was 0.70, with a sensitivity of 0.78 and specificity of 0.60. Tau showed higher discriminability for mTBI+ patients and TCs (AUC = 0.80, sensitivity = 0.79, specificity = 0.85).

In the analysis including GFAP within 72 h, tau within 72 h, NFL at 2 weeks and age, the best-glm separating patients with mTBI and combined controls yielded a model including age, GFAP measured within 72 h and NFL at 2 weeks. The combined AUC of this model was 0.80, with a sensitivity of 0.53 and specificity of 0.94. The same model best-glm comparing mTBI+ to TCs yielded a model including the same variables of age, GFAP measured within 72 h and NFL at 2 weeks. The combined AUC was 0.85, with a sensitivity of 0.65 and high specificity of 0.98.

Lastly, the best-subsets model conducted using a subset of patients with uncomplicated mTBI vs. controls yielded a model including age, GFAP measured within 72 h and NFL at 2 weeks. The combined AUC was 0.79, the sensitivity was 0.60 and specificity was 0.85.

## Discussion

In this large prospective study of a representative group of patients with mixed-mechanism mTBI and matched control groups (32, 33), we demonstrated different temporal trajectories, associations and discriminative powers of the CNS-associated injury markers GFAP, NFL and tau in peripheral blood. Moreover, we were able to assess the role of other somatic injuries with and without an mTBI on GFAP, NFL and tau levels in blood by stratifying patients with mTBI into groups with and without other injures and comparing them to both CC and TC groups.

### Longitudinal Evolution of Blood Biomarkers From the Acute Phase to 12 Months Post-Injury

Glial fibrillary acidic protein levels showed the largest differences from the combined control group in the acute phase. The difference was reduced, but still significant at 72-h, and patient levels decreased thereafter, becoming comparable to controls by 2 weeks. Our results extend prior mTBI studies by showing that GFAP is most significantly elevated within 24 h following injury, remains elevated for a number of days, but returns to control levels by ~2 weeks (4, 11, 12, 17).

NFL concentrations did not differ from controls acutely or at 72-h, but peaked at 2 weeks, remaining significantly elevated at 3 months and returning to control levels by 12 months. NFL's peak at 2 weeks and significant elevation at 3 months concurs with the sports concussion literature (22, 23). We show for the first time that NFL levels return to control levels by 12 months in patients with mixed-mechanism mTBI.

Tau levels were elevated compared to controls only in the acute phase, in line with results from the literature on both sports concussion (23, 27) and mixed-mechanism TBI (9). Our findings emphasize the importance of early sampling of plasma tau. Thereafter, there was considerable variability in tau blood levels, suggesting differences in the evolution of neuronal pathology over time among patients with mTBI. S100 has been shown to exhibit a second peak as a result of ongoing astroglial damage (7). There may be a similar process occurring with tau, leading to the observed temporal variability in tau levels. However, we were not able to demonstrate a significant difference from controls at any later timepoint in our sample, thus the clinical significance of this in mTBI may be understated.

The linear mixed model comparing mTBI– to community controls showed similar results to those mentioned above, indicating the demonstrated effects cannot be attributed to the potential confound of somatic injuries.

The rapid increases in both tau and GFAP in blood within 24 h after TBI could stem from acute injury to neuronal and astroglial cells, respectively, combined with an opening of the blood-brain barrier due to trauma. Tau may also originate from the extracellular space (39). The delayed NFL elevation probably reflects pathophysiological processes associated with progressive secondary axonal injury (40). The protracted period of NFL elevation in mTBI, and can reflect ongoing secondary CNS injury, and makes it a potentially interesting biomarker for assessing duration and severity of secondary pathology in mTBI beyond the acute stage. However, neurofilament proteins have been associated with axonal regeneration in degenerative diseases (41), thus increased levels following mTBI could reflect ongoing CNS repair mechanisms. Lastly, given the high correlation between acutely measured GFAP and tau with subacute NFL, these biomarkers might reflect similar injury mechanisms but at different stages in time. In summary, the protracted increase in peripheral NFL levels, allows for a longer time widow for obtaining peripheral plasma biomarkers of mTBI in the clinic, represent ongoing CNS injury and/or repair processes, likely linked to the early injury as reflected by acute GFAP and tau levels.

### The Demographic and Clinical Variables Most Predictive of Biomarker Concentrations

In the best-subset analyses, each studied biomarker was differentially associated with injury-related variables (traumatic intracranial MRI findings, somatic injuries, GCS scores, LOC, and PTA) and the demographic variables sex and age.

Importantly, the models for each of the three biomarkers included presence of positive intracranial traumatic MRI findings at all selected timepoints. Glial fibrillary acidic protein levels have previously been associated with traumatic intracranial findings in mTBI (4, 9, 12, 17). Our results demonstrated larger effect sizes at the acute timepoint than at 72-h, indicating GFAP's usefulness in predicting traumatic intracranial injury decreases sharply after injury, and reiterating the notion that GFAP reflects acute injury. Our findings show that NFL provides useful clinical information regarding the presence of traumatic intracranial findings in patients with mTBI at later timepoints. A previous study sampling NFL within 48 h also showed higher NFL levels in those with traumatic intracranial findings as measured by MRI/CT (12). We are the first to demonstrate these effects at 2 weeks and 3 months post-injury. Tau measured acutely was also significantly associated with intracranial findings on MRI, though the effect was much smaller than that found for GFAP or NFL. Tau's utility as a marker of intracranial injury is thus limited, in line with prior literature (12).

Glasgow Coma Scale score was included in the best models for 72-h GFAP and NFL at 2 weeks and 3 months. Glasgow Coma Scale score's inclusion in the GFAP model indicates that lower GCS score may be related to greater astrocytic injury, while associations with NFL suggest that lower GCS scores are related to more serious and protracted secondary axonal injury. Furthermore, LOC was included in 72-h GFAP and 3-month NFL models (42). Many theories of altered consciousness following mTBI implicate damage to axons. That an effect of LOC was found for NFL only when measured at 3 months, suggests that longer periods of unconciousness at time of injury predict greater protracted release of NFL, potentially reflecting greater ongoing secondary axonal injury.

The best models for GFAP and NFL both included presence of somatic injuries, at all timepoints. This is in line with GFAP and NFL being more elevated in the mTBI+ than the mTBI– group. It might be that GFAP and NFL levels are higher in those injured in traffic accidents or by violence due to for instance sustaining a higher energy impact. However, based on our other measured clinical variables, we were unable to demonstrate greater CNS involvement in the mTBI+ group, or that GFAP and NFL are released from areas in the body other than the CNS. This relationship is further explored in the next section.

Age was included in the best models of both GFAP and NFL. During aging, there are a number of cellular changes leading to increased neural susceptibility to damage (43). It is believed that the effects of CNS disease-related processes are amplified in aging neural cells (44), which may be reflected in higher plasma biomarker levels during TBI.

Sex was included as a predictor in the best model for acute tau, with women exhibiting higher tau levels following mTBI than men. This could be due to genetic sex differences in tau expression causing men to underexpress tau (45).

The above analyses were repeated on a subset of patients with mTBI without intracranial traumatic findings on MRI (i.e., uncomplicated mTBI). The goal was to ascertain whether the clinical and demographic associations determined previously held up in this category of patients with mTBI. The results were quite similar for the complicated and uncomplicated mTBI groups. However, a fundamental difference was that LOC was included in the models for 72-h GFAP, and 2-week and 3-month NFL for the uncomplicated mTBI group while GCS score was included in the model for those with complicated mTBI. This suggests that both GCS and LOC may reflect similar injury characteristics, but that GCS score is likely more diagnostically relevant in patients with complicated mTBI (i.e., with presence of intracranial traumatic findings), while LOC is more relevant in milder cases. Moreover, somatic injuries were included as a predictor in the acute tau model in uncomplicated mTBI, while they were not considered an important predictor in the acute tau model with all subjects included, whose predictors were sex and MRI findings. This may indicate that bodily injuries are of some importance in tau release (see below), but that this importance is eclipsed by MRI findings, when they are present.

### The Effect of Other Somatic Injuries on Plasma Biomarker Levels

There was no evidence for elevated NFL, GFAP or tau levels in the TC compared to the CC group in any of the comparisons. This observation supports the premise that these plasma biomarkers are CNS specific, however it should be noted that TC only had blood data available at acute and chronic timepoints, leaving open the possibility that there may be a difference at subacute timepoints. Also, there were few samples from the TC group in the acute-72-h period. Still, for all biomarkers and at all timepoints, the mTBI+ group had higher levels of NFL, GFAP, or tau than the mTBI– group. Given the results of our mTBI+ vs. mTBI– group comparisons, the higher GFAP, NFL, and tau levels cannot be explained by a higher frequency of intracranial findings on brain MRI, more severe brain injury as reflected in GCS score, presence of LOC or duration of PTA, or be related to intoxication at time of injury. Injury mechanism might play a role, as there was a significant overall difference in the frequency of injury type between the mTBI+ and mTBI– groups, with the mTBI+ group more likely to be involved in a traffic accident than the mTBI– group. The differences in injury mechanisms between the TC and mTBI groups were more striking. The mTBI+ group was significantly more likely to be injured in a traffic accident or due to violence than the TC group. Also, the mTBI– group was more often injured in traffic accidents than TC. On the other hand, the TC group suffered more sports related injures than both the mTBI+ and mTBI– groups. Thus, those in the mTBI+ group may experience more high impact injuries and consequently greater neural damage than those in the mTBI– and the TC group. These differences, however, were not reflected by the clinical or neuroradiological examinations.

The Human Protein Atlas (www.proteinatlas.org) (46) reports that NFL, GFAP, and tau are expressed in small amounts in tissues such as muscle and soft tissues. It is possible the injury types more frequently experienced by those in the mTBI+ group, combined with the more frequent higher energy injury mechanism, led to additional release of these proteins from muscle and soft tissues. Nevertheless, the mTBI+ group was always more different from the TC group than the mTBI– group, supporting the notion that patients with mTBI and additional somatic injuries (mTBI+) represent a distinct group that differs from both the mTBI– and TC groups. The mechanisms behind and clinical significance of the higher blood biomarker levels in the mTBI+ group require further study.

### Biomarkers' Discriminability Between Patients With mTBI and Controls

Only GFAP and NFL discriminated mTBI from the combined control group at a level which could be clinically relevant. Glial fibrillary acidic protein had very good discriminability at both early timepoints, confirming previous findings and emphasizing the importance of early GFAP sampling in mTBI (9, 12). Glial fibrillary acidic protein showed a similarly strong ability to discriminate mTBI+ from TCs with lower sensitivities but higher specificities. This indicates that GFAP correctly identifies trauma controls from the mTBI+ group better than controls from all patients with mTBI.

NFL showed moderate ability to classify mTBI patients from combined controls at both 2 weeks and 3 months. Sensitivity at both timepoints was quite high, while specificity was low, indicating there was a large degree of misclassification of controls as patients. A previous mixed-mechanism mTBI (9) and a sports concussion study on boxers (22) reported higher AUCs in the first week after injury comparable to what we found at 2 weeks. The discrepancy may be due to the other studies recruiting mTBI patients only from a hospital ED, purportedly reflecting more severe injury, and that boxers likely experience more high-energy trauma, which is associated with increased axonal damage (47). We recruited from both general practitioners and hospital EDs, with patients from the general practitioners ED on average having a higher GCS score (33) putatively reflecting less severe injury. Conversely, NFL showed extremely high specificity, but low sensitivity in distinguishing mTBI+ patients from TCs. Similar to GFAP, this shows that NFL can discriminate trauma controls from mTBI+ patients better than controls from all patients with mTBI. This provides further evidence that the characteristics of mTBI+ patients differ from other groups and supports the notion that high-energy trauma is more likely to increase both NFL and GFAP levels.

Most of the patients with mTBI exhibited tau levels within control ranges, thus the efficacy of plasma tau in discriminating between mTBI and controls is eclipsed by GFAP and NFL. In a prior mixed-mechanism TBI study, plasma tau measured within 48 h showed similar AUC (0.66) (9) to our acute (within 24 h) timepoint (AUC = 0.70). We propose their reported slightly lower discriminatory power is due to the longer time period of sampling. In the present study, tau showed a greater ability to discriminate mTBI+ from TCs (AUC = 0.80). Unlike GFAP and NFL, both sensitivity and specificity were adequate (0.79 and 0.85, respectively), indicating acute tau may be appropriate for distinguishing mTBI+ patients from controls. This finding reiterates that the mTBI+ group differs from the mTBI– group, and this difference needs to be studied further for a better understanding of mTBI pathophysiology and outcome when other injuries are present.

A best-subsets logistic regression was performed to determine the best combination of multiple blood biomarkers at different timepoints as well as demographic variables to discriminate all patients with mTBI from controls, and the mTBI+ group from TC group. The models for both had the same variables included and yielded very high specificities (0.94 and 0.98, respectively) but low sensitivities (0.53 and 0.65, respectively). This suggests that both models correctly identify controls with a high degree of accuracy but were unable to correctly identify patients. Based on the data in our sample, the combined use of age, acutely measured GFAP and protracted NFL is able to correctly identify almost all controls but would miss a large number of patients with less severe injuries. A similarly low sensitivity and high specificity was found in the best model discriminating patients with uncomplicated mTBI (no intracranial findings on MRI) from controls, leading to similar conclusions as for the whole mTBI group.

In summary, the blood biomarkers GFAP measured in the early phase and NFL obtained in the subacute to 3-month period were both able to discriminate between individuals sustaining a mTBI from the control groups. No added value of improved mTBI discrimination was achieved by combining early GFAP and late NFL in the model.

### Strengths and Limitations

Our study has a number of strengths compared to prior studies. We screened every eligible person who presented to both hospital and general practitioner-run EDs with blunt head trauma and clinical symptoms indicating a diagnosis of mTBI according to WHO/ICD10 criteria, and enrolled a high percentage of eligible patients. These were followed longitudinally for 1 year, with minimal attrition (Figure 1), along with two age- and sex- matched control groups. Given the wide spectrum of injury mechanisms, along with a large sample size, our study allows for greater generalizability than previous studies, which are typically underpowered or focus on specific mTBI cohorts, such as sports concussion.

There are also some limitations. Acute and 72-h samples were not often drawn from the same individuals (see Figure 1), leading to relatively independent samples at these timepoints. For TCs, those who agreed to participate in the study had usually left the clinic before early blood sampling could be performed, meaning few had blood drawn at acute and 72-h timepoints, but many at 3 months. CC also did not have blood drawn at 72 h or 2 weeks, leading to small control sample sizes at these two timepoints (see Supplementary Tables 9–11 for further details). Age < 60 years was an inclusion criterion in this study chosen to reduce the burden of age-related findings on brain MRI. The effect of age beyond 60 years on blood biomarker levels can therefore not be determined in this study. Lastly, future research should assess the prognostic utility of the biomarkers and injury-related variables assessed in this study on functional outcome, such as post-concussion syndrome.

### Clinical Implications and Concluding Remarks

The three biomarkers measured in this study all satisfied different aspects of the desired qualifications marking them as useful mTBI biomarkers. All were significantly associated with traumatic intracranial MRI findings, demonstrating their association with more severe CNS damage. Each had a unique temporal profile and were differentially related to injury characteristics. Presence of other somatic injury in combination with mTBI was associated with greater biomarker elevation and duration, suggesting that certain injury mechanisms are related to greater release of both acute (GFAP and tau) and secondary injury-related (NFL) CNS biomarkers. Taken together, the different biomarkers could be used to determine severity of both primary and secondary injury, and could become objective proxies of clinical mTBI characteristics.

Glial fibrillary acidic protein appears to be most useful during the acute phase. NFL, with its late peak and significant associations with MRI findings at later timepoints, could aid clinicians in monitoring the severity of secondary injury. We propose NFL could allow for a longer diagnostic time window and aid in the monitoring of secondary axonal injury, which may be useful for assisting sports-physicians in return-to-play decisions and in assessing possible long-term symptoms following concussion in contact-sports athletes. Peripheral blood measures of tau were weakly associated with MRI findings, and provided moderate discriminability of all patients with mTBI from controls if measured acutely. Plasma tau appears to be a less useful plasma biomarker than GFAP or NFL, however our results indicate it may be appropriate for distinguishing the subclass of patients with mTBI and other somatic injuries from controls. Finally, the temporal evolution of the plasma biomarkers of neural injury across 1 year as well as the biomarkers concentrations' associations with demographic and clinical variables, can inform the interpretation of these biomarkers in other neurological disorders where they are elevated (48).

## Data Availability Statement

The raw data supporting the conclusions of this article will be made available by the authors, without undue reservation.

## Ethics Statement

The studies involving human participants were reviewed and approved by Mid-Norway Regional Committee for Research Ethics (REK 2013/754). Written informed consent to participate in this study was provided by the participants' legal guardian/next of kin.

## Author Contributions

GC organized blood data results, performed all statistical analyses, and drafted the manuscript for intellectual content. TS (PI of Trondheim mild TBI study), AV, and AH designed the study, oversaw all data collection, contributed to analysis, planned, and revised the manuscript. HZ in collaboration with KB, picked out and oversaw blood analysis, quality assessed data, and revised the manuscript. CE was involved in recruitment of participants, collected and organized demographic, clinical data and blood sample data, organized MRI data files, provided data files, and revised the manuscript. CE and CF recruited participants, collected and processed blood samples, organized variable files, and revised the manuscript. TF is the statistician who approved all statistical methods and presentation of results in writing and figures/tables. AV designed the study with TS and AH, provided neurosurgical expertise, and revised the manuscript. KB was the main investigator responsible for blood analyses and quality approval, provided input to the analysis, and revised the manuscript. AH is the principal supervisor of this manuscript, planned the analyses, advised GC, planned, and revised the manuscript. All authors contributed to the article and approved the submitted version.

## Conflict of Interest

HZ is a Wallenberg Academy Fellow supported by grants from the Swedish Research Council (#2018-02532), the European Research Council (#681712) and Swedish State Support for Clinical Research (#ALFGBG-720931). KB holds the Torsten Söderberg Professorship in Medicine at the Royal Swedish Academy of Sciences, and was supported by the Swedish Research Council (#2017-00915), the Swedish Alzheimer Foundation (#AF651 742881), Hjärnfonden, Sweden (#FO2017-0243), and a grant (#ALFGBG-715986) from the Swedish state under the agreement between the Swedish government and the County Councils, the ALF-agreement. KB has served as a consultant or at advisory boards for Alzheon, CogRx, Biogen, Lilly, Novartis and Roche Diagnostics, and is a co-founder of Brain Biomarker Solutions in Gothenburg AB, a GU Venture-based platform company at the University of Gothenburg. The remaining authors declare that the research was conducted in the absence of any commercial or financial relationships that could be construed as a potential conflict of interest.

## Supplementary Material

Supplementary Figure 1 Concentrations of GFAP **(A)**, NFL **(B)**, and tau **(C)** over time in patients with mTBI and combined control group. Data are presented as box plots with median as the midline, box borders representing the 25th and 75th percentile and whiskers calculated as the 25th and 75th percentile + 1.5 ^*^ interquartile range. Points above and below the whiskers represent outliers. Individual data points are presented within the box-plots. GFAP and NFL are presented on a log-transformed scale, while tau retains its original scale, for visualization purposes. Asterisks (^*^) indicate that the mixed model demonstrated a significant group difference between patients with mTBI and controls at the indicated timepoint. The *p*-value level is represented as follows: ^***^*p* < 0.001, ^****^*p* < 0.0001. GFAP, Glial fibrillary acidic protein; NFL, Neurofilament light.

Supplementary Table 1 Results of a Mann-Whitney *U*-test of differences in biomarker concentrations between trauma and community controls at acute and 3-month time points.

Supplementary Table 2 Summary of main effects of group (mTBI patients vs. controls), timepoint and the interaction of group and time, for each of the 3 biomarkers, based on a linear mixed model.

Supplementary Table 3 Summary of main effects of group [patients with mTBI without somatic injuries (mTBI–) vs. community controls], timepoint and the interaction of group and time, for each of the 3 biomarkers, based on a linear mixed model.

Supplementary Table 4 Main effects of the one-way between subjects ANOVA comparing mTBI+, mTBI–, TC, and CC for each of the 3 biomarkers at chosen timepoints.

Supplementary Table 5 Results of all-subsets multiple linear regression with each of the biomarkers as outcome variables and all demographic and clinical variables listed at the top of the table as possible predictors.

Supplementary Table 6 Means and standard deviations of biomarkers at chosen timepoints, grouped by dichotomous injury-related and demographic characteristics.

Supplementary Table 7 ROC analysis results on the discriminability of biomarkers at chosen timepoints for patients with mTBI vs. controls.

Supplementary Table 8 ROC analysis results on the discriminability of biomarkers at chosen timepoints for patients with mTBI and other somatic injuries (mTBI+) vs. trauma controls.

Supplementary Table 9 Descriptive statistics of GFAP, NFL, and tau concentrations, on their original scale, in patients with mTBI and controls, at each timepoint.

Supplementary Table 10 Descriptive statistics of GFAP, NFL, and tau concentrations, on their original scale, in patients with mTBI and other somatic injuries (mTBI+) and patients without other somatic injuries (mTBI–), at each timepoint.

Supplementary Table 11 Descriptive statistics of GFAP, NFL, and tau concentrations, on their original scale, in community controls and trauma controls, at each timepoint.
