# Supplementary figures and images for: One-Year Prospective Study of Plasma Biomarkers From CNS in Patients With Mild Traumatic Brain Injury
Source: Front Neurol. 2021 Apr 21;12:643743. doi: 10.3389/fneur.2021.643743 (PMC8097004; doi:10.3389/fneur.2021.643743)

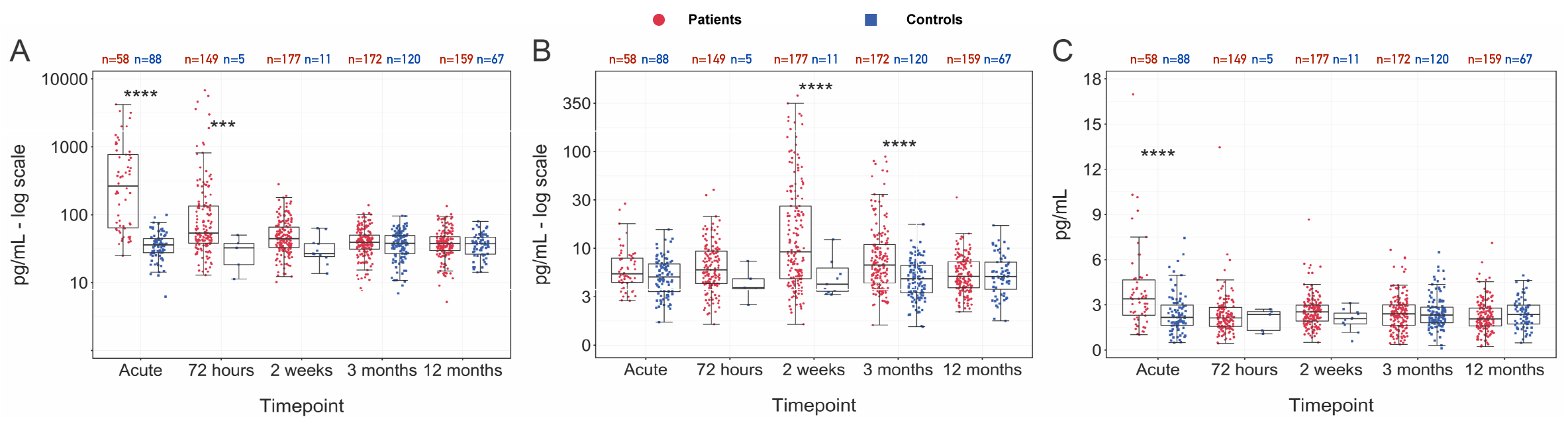

Supplement: Supplementary Figure 1 — Concentrations of GFAP (A), NFL (B), and tau (C) over time in patients with mTBI and combined control group. Data are presented as box plots with median as the midline, box borders representing the 25th and 75th percentile and whiskers calculated as the 25th and 75th percentile + 1.5 * interquartile range. Points above and below the whiskers represent outliers. Individual data points are presented within the box-plots. GFAP and NFL are presented on a log-transformed scale, while tau retains its original scale, for visualization purposes. Asterisks (*) indicate that the mixed model demonstrated a significant group difference between patients with mTBI and controls at the indicated timepoint. The p-value level is represented as follows: ***p < 0.001, ****p < 0.0001. GFAP, Glial fibrillary acidic protein; NFL, Neurofilament light. [file Image_1.TIF]
