# Supplementary material for: One-Year Prospective Study of Plasma Biomarkers From CNS in Patients With Mild Traumatic Brain Injury
Source: Front Neurol. 2021 Apr 21;12:643743. doi: 10.3389/fneur.2021.643743 (PMC8097004; doi:10.3389/fneur.2021.643743)
Supplement: Supplementary Table 1 — Results of a Mann-Whitney U-test of differences in biomarker concentrations between trauma and community controls at acute and 3-month time points. [file Data_Sheet_1.docx]

**Supplementary Table 1:** Results of a Mann-Whitney U test of differences in biomarker concentrations between trauma and community controls at acute and 3-month time points.

|  | **Acute** | | | **3 months** | | |
| --- | --- | --- | --- | --- | --- | --- |
|  | GFAP | NFL | Tau | GFAP | NFL | Tau |
| U statistic | 248 | 249 | 310 | 1687 | 1874 | 1793 |
| *p*–value | .980 | .967 | .293 | .938 | .355 | .625 |

GFAP, Glial fibrillary acidic protein; NFL, Neurofilament light.

**Supplementary Table 2**. Summary of main effects of group (mTBI patients versus controls), timepoint and the interaction of group and time, for each of the 3 biomarkers, based on a linear mixed model.

| **Biomarkers** | **Group**  F-value (*p-value*) | **Time point**  F-value (*p-value*) | **Interaction (Group x Time)**  F-value (*p-value*) |
| --- | --- | --- | --- |
| GFAP | F = 46.67, ***p* < .0001** | F = 84.99, ***p* < .0001** | F = 70.22, ***p* < .0001** |
| NFL | F = 20.24, ***p* < .0001** | F = 84.89, ***p* < .0001** | F = 9.72, ***p* < .0001** |
| Tau | F = 1.53, *p* = .218 | F = 7.78, ***p* < .0001** | F = 11.18, ***p* < .0001** |

All biomarker values are log transformed. P-values in bold indicate statistical significance.

GFAP, Glial fibrillary acidic protein; NFL, Neurofilament light.

**Supplementary Table 3**. Summary of main effects of group (patients with mTBI without somatic injuries (mTBI-) versus community controls), timepoint and the interaction of group and time, for each of the 3 biomarkers, based on a linear mixed model.

| **Biomarkers** | **Group**  F-value (*p-value*) | **Timepoint**  F-value (*p-value*) | **Interaction (Group x Time)**  F-value (*p-value*) |
| --- | --- | --- | --- |
| GFAP | F = 26.54, ***p* < .0001** | F = 38.26, ***p* < .0001** | F = 112.26, ***p* < .0001** |
| NFL | F = 4.85, ***p* = .029** | F = 8.33, ***p* = .0003** | F = 8.91, ***p* = .0002** |
| Tau | F = 0.01, *p* = .907 | F = 2.53, *p* = .082 | F = 8.90, ***p* = .0002** |

All biomarker values are log transformed. P-values in bold indicate statistical significance.

GFAP, Glial fibrillary acidic protein; NFL, Neurofilament light.

**Supplementary Table 4**. Main effects of the one-way between subjects ANOVA comparing mTBI+, mTBI-, TC and CC for each of the 3 biomarkers at chosen timepoints.

| **Biomarkers** | **F-value** | **P- value** |
| --- | --- | --- |
| GFAP - Acute | 55.21 | **<.0001** |
| GFAP - 72 hours | 5.20 | **.007** |
| NFL - 2 weeks | 12.00 | **<.0001** |
| NFL - 3 months | 16.38 | **<.0001** |
| Tau - Acute | 8.36 | **<.0001** |

All biomarker values are log transformed. P-values in bold indicate statistical significance.

GFAP, Glial fibrillary acidic protein; NFL, Neurofilament light.

**Supplementary Table 5.** Results of all-subsets multiple linear regression with each of the biomarkers as outcome variables and all demographic and clinical variables listed at the top of the table as possible predictors.

|  | β-value [95% CI]  *p*-value | | | | | | |
| --- | --- | --- | --- | --- | --- | --- | --- |
|  | Sex: Female^1^ | Age | GCS: 13-14^2^ | LOC: Observed^3^ | PTA: 1-24 hours^4^ | MRI Finding: Yes^5^ | Somatic Injuries: Yes^6^ |
| GFAP: Acute |  |  |  |  |  | 0.94 [0.55 – 1.33]  ***p* < .0001** | 0.29 [0.02 – 0.56]  ***p* =** **.039** |
| GFAP: 72 hours |  | 0.01 [0.006 – 0.02]  ***p* = .0002** | 0.19 [-0.03 – 0.40]  *p* = .088 | 0.12 [-0.04 – 0.29]  *p* = .141 |  | 0.57 [0.30 – 0.84]  ***p* < .0001** | 0.22 [0.05 – 0.39]  ***p* = .013** |
| NFL: 2 weeks |  | 0.009 [0.004 – 0.01]  ***p* = .0008** | 0.23 [0.04 – 0.41]  ***p* = .017** |  |  | 0.77 [0.55 – 0.99]  ***p* <.0001** | 0.27 [0.13 – 0.42]  ***p* = .0002** |
| NFL: 3 months |  | 0.006 [0.003 – 0.01]  ***p* = .0004** | 0.11 [-0.01 – 0.23]  *p* =.080 | 0.12 [0.03 – 0.21]  ***p* = .01** |  | 0.47 [0.33 – 0.61]  ***p* <.0001** | 0.12 [0.03 – 0.21]  ***p* = .009** |
| Tau: Acute | 0.17 [0.04 – 0.30]  ***p* = .012** |  |  |  |  | 0.18 [0.002 – 0.37]  ***p* = .048** |  |

Empty spaces indicate the best model fit did not include that association. All biomarker values were log-transformed prior to inclusion into the model. Beta values are unstandardized. Significant p-values (*p* < .05) are bolded. GFAP, Glial fibrillary acidic protein, NFL, Neurofilament light; GCS, Glasgow coma score; LOC, Loss of consciousness; PTA, Post-traumatic amnesia; MRI, Magnetic resonance imaging; 95% CI, 95% confidence interval.

^1^ Baseline comparison for Sex was male.

^2^ Baseline comparison for GCS scores of 13-14 was GCS score of 15.

^3^ Baseline comparison for LOC observed was "No LOC observed"

^4^ Baseline comparison for PTA duration of between 1–24 hours was PTA of less than 1 hour.

^5^ Baseline comparison for MRI findings was "No MRI finding".

^6^ Baseline comparison for somatic injuries was “no other somatic injuries”.

|  | **Sex:** | | **GCS:** | | **LOC:** | | **PTA:** | | **MRI Finding:** | | **Somatic Injuries:** | | |
| --- | --- | --- | --- | --- | --- | --- | --- | --- | --- | --- | --- | --- | --- |
|  | Male | Female | 15 | 13-14 | Not observed | Observed | < 1 hour | 1-24 hours | No | Yes | No | Yes |  |
| **GFAP**: Acute | 574.3 ± 816.4 | 730.9 ± 1030.4 | 571.9 ± 833.3 | 859.6 ± 1117.0 | 434.2 ± 631.1 | 885.2 ± 1115.3 | 510.5 ± 763.4 | 1072.3±1208.9 | 387.7 ± 480 | 2190.8±1344.4 | 495.5 ± 912.3 | 836.1 ± 866.4 |  |
| **GFAP**: 72 hours | 253.5 ± 689.0 | 360.2 ±1195.5 | 281.7 ± 882.6 | 436.9±756.9 | 227.4 ± 1035.2 | 288.8 ± 296.7 | 288.8 ± 856.6 | 296.7 ± 988.7 | 208.6 ± 691.0 | 1089.9±1880.5 | 215.7 ± 743.0 | 432.6 ± 1130.8 |  |
| **NFL**: 2 weeks | 31.7 ± 55.3 | 31.7 ± 60.4 | 26.8 ± 45.2 | 64.6 ± 98.6 | 26.1 ± 53.5 | 37.5 ± 60.2 | 25.1 ± 42.9 | 47.3 ± 79.4 | 23.1 ± 42.4 | 108.1 ± 100.4 | 23.9 ± 55.6 | 45.4 ± 57.4 |  |
| **NFL**: 3 months | 11.4 ± 13.6 | 11.6 ± 16.4 | 10.6 ± 12.1 | 17.9 ± 23.7 | 8.5 ± 10.1 | 14.5 ± 17.6 | 10.0 ± 10.8 | 14.8 ± 20.6 | 9.2 ± 10.7 | 31.1 ± 25.5 | 10.4 ± 15.4 | 13.3 ± 13.0 |  |
| **Tau**: Acute | 3.3 ± 2.2 | 4.9 ± 3.4 | 4.0 ± 3.0 | 3.8 ± 2.0 | 4.2 ± 3.2 | 3.6 ± 2.2 | 3.8 ± 2.9 | 4.6 ± 2.5 | 3.7 ± 2.7 | 5.5 ± 2.8 | 3.6 ± 3.0* | 4.4 ± 2.5* |  |

**Supplementary Table 6.** Means and standard deviations of biomarkers at chosen timepoints, grouped by dichotomous injury-related and demographic characteristics.

Age was not included as it is not a dichotomous variable. Colored backgrounds correspond to those in Figure 3, i.e. all colored backgrounds are predictors included in the best model, as assessed using the best-model multiple regression, with each biomarker input as an outcome, and all other variables included as possible predictors. Biomarker values retain their original scale. A white background indicates that the predictor was not included in the final model. The direction and size of regression coefficient are represented according to the figure legend color scheme shown in Figure 3, whereby increasingly positive associations are graded to red.

GFAP, Glial fibrillary acidic protein, NFL, Neurofilament light; GCS, Glasgow coma score; LOC, Loss of consciousness; PTA, Post-traumatic amnesia; MRI, Magnetic resonance imaging.

**Supplementary Table 7:** ROC analysis results on the discriminability of biomarkers at chosen timepoints for patients with mTBI versus controls.

|  | **Best Method**^‡^ | | |  |
| --- | --- | --- | --- | --- |
|  | **Sensitivity** | **Specificity** | **Threshold** (pg/mL) | **AUC** [95% CI] |
| **GFAP:** |  |  |  |  |
| Acute | 0.88 | 0.81 | 57.50 | 0.92 [0.88 – 0.97] |
| 72 hours | 0.67 | 0.72 | 41.09 | 0.74 [0.69 – 0.80] |
| **NFL:** |  |  |  |  |
| 2 weeks | 0.93 | 0.52 | 8.88 | 0.75 [0.70 – 0.80] |
| 3 months | 0.85 | 0.45 | 7.44 | 0.68 [0.62 – 0.74] |
| **Tau:** |  |  |  |  |
| Acute | 0.78 | 0.60 | 3.00 | 0.70 [0.62 – 0.79] |
| **Best-Subset*** | 0.53 | 0.94 | / | 0.80 [0.76 – 0.85] |

^‡^ Best Method is defined by Youden’s J statistic, in which the optimal threshold that maximizes both sensitivity and specificity is calculated.

* Best-subset model: Possible predictors fed in the model were Age, GFAP measured within 72 hours, Tau measured within 72 hours and NFL measured at 2 weeks. The best model fit included Age, GFAP and NFL, but not Tau.

mTBI, mild traumatic brain injury; GFAP, Glial fibrillary acidic protein; NFL, Neurofilament light; ROC, Receiver Operating Characteristic; AUC, Area Under the Curve; 95% CI is the 95% confidence interval of the estimated AUC.

**Supplementary Table 8:** ROC analysis results on the discriminability of biomarkers at chosen timepoints for patients with mTBI and other somatic injuries (mTBI+) versus trauma controls.

|  | **Best Method**^‡^ | | |  |
| --- | --- | --- | --- | --- |
|  | **Sensitivity** | **Specificity** | **Threshold** (pg/mL) | **AUC** [95% CI] |
| **GFAP:** |  |  |  |  |
| Acute | 0.83 | 0.94 | 70.07 | 0.92 [0.85 – 0.99] |
| 72 hours | 0.56 | 0.94 | 68.76 | 0.80 [0.72 – 0.89] |
| **NFL:** |  |  |  |  |
| 2 weeks | 0.66 | 1.00 | 10.25 | 0.83 [0.75 – 0.91] |
| 3 months | 0.60 | 0.88 | 7.77 | 0.78 [0.70 – 0.87] |
| **Tau:** |  |  |  |  |
| Acute | 0.79 | 0.85 | 2.97 | 0.80 [0.68 – 0.92] |
| **Best-Subset*** | 0.65 | 0.98 | / | 0.85 [0.79 – 0.93] |

^‡^ Best Method is defined by Youden’s J statistic, in which the optimal threshold that maximizes both sensitivity and specificity is calculated.

* Best-subset model: Possible predictors fed in the model were Age, GFAP measured within 72 hours, Tau measured within 72 hours and NFL measured at 2 weeks. The best model fit included Age, GFAP and NFL, but not Tau.

mTBI, mild traumatic brain injury; GFAP, Glial fibrillary acidic protein; NFL, Neurofilament light; ROC, Receiver Operating Characteristic; AUC, Area Under the Curve; 95% CI is the 95% confidence interval of the estimated AUC.

|  | **Patients with mTBI** | | | | | **Combined Controls** | | | | |
| --- | --- | --- | --- | --- | --- | --- | --- | --- | --- | --- |
|  | **Acute**  N = 58 | **72 hours**  N = 149 | **2 weeks**  N = 177 | **3 months**  N = 172 | **12 months**  N = 159 | **Acute**  N =88 | **72 hours**  N = 5 | **2 weeks**  N = 11 | **3 months**  N = 120 | **12 months**  N = 67 |
| **GFAP (pg/mL)** |  |  |  |  |  |  |  |  |  |  |
| Mean | 636.4 | 291.4 | 54.9 | 43.2 | 41.7 | 38.2 | 30.1 | 33.1 | 39.5 | 37.6 |
| Median | 266.2 | 53.7 | 44.3 | 39.5 | 37.9 | 35.8 | 32.5 | 26.7 | 38.1 | 37.5 |
| SD | 901.9 | 899.7 | 37.0 | 21.2 | 20.9 | 17.4 | 15.5 | 16.2 | 17.4 | 14.6 |
| IQR | 702.5 | 96.3 | 32.8 | 18.9 | 17.8 | 16.8 | 19.4 | 13.5 | 22.8 | 20.4 |
| Range | 25.0–4212.9 | 12.9–6815.1 | 10.2–281.5 | 7.8–138.4 | 5.19–133.9 | 6.2–100.0 | 11.3–50.3 | 13.6–63.5 | 7.0–96.3 | 14.2–80.2 |
| **NFL (pg/mL)** |  |  |  |  |  |  |  |  |  |  |
| Mean | 7.0 | 7.5 | 31.7 | 11.5 | 5.9 | 5.6 | 4.5 | 5.4 | 5.3 | 5.8 |
| Median | 5.5 | 6.0 | 9.2 | 6.7 | 5.1 | 5.1 | 3.9 | 4.2 | 4.8 | 5.1 |
| SD | 4.8 | 5.4 | 57.0 | 14.6 | 3.3 | 2.6 | 1.8 | 2.7 | 2.5 | 2.9 |
| IQR | 3.5 | 5.1 | 22.5 | 6.6 | 3.4 | 3.3 | 1.0 | 2.7 | 3.2 | 3.4 |
| Range | 2.9–29.0 | 1.6–40.3 | 1.6–379.2 | 1.6–88.5 | 2.2–33.7 | 1.7–15.6 | 2.6–7.4 | 3.3–12.4 | 1.5–17.7 | 1.8–17.3 |
| **Tau (pg/mL)** |  |  |  |  |  |  |  |  |  |  |
| Mean | 4 | 2.3 | 2.6 | 2.5 | 2.2 | 2.4 | 2.0 | 2.0 | 2.5 | 2.4 |
| Median | 3.4 | 2.1 | 2.5 | 2.4 | 2.1 | 2.2 | 2.4 | 2.1 | 2.3 | 2.4 |
| SD | 2.8 | 1.4 | 1.0 | 1.1 | 1.1 | 1.3 | 0.8 | 0.7 | 1.1 | 1.0 |
| IQR | 2.3 | 1.3 | 1.1 | 1.3 | 1.2 | 1.3 | 1.2 | 0.7 | 1.0 | 1.2 |
| Range | 1.0–17.0 | 0.4–13.5 | 0.5–8.7 | 0.4–6.6 | 0.2–7.1 | 0.5–7.4 | 1.1–2.7 | 0.6–3.1 | 0.1–6.5 | 0.5–5.0 |

**Supplementary Table 9:** Descriptive statistics of GFAP, NFL and tau concentrations, on their original scale, in patients with mTBI and controls, at each timepoint.

mTBI, mild traumatic brain injury; GFAP, Glial fibrillary acidic protein; NFL, Neurofilament light.

|  | **mTBI+** | | | | | **mTBI-** | | | | |
| --- | --- | --- | --- | --- | --- | --- | --- | --- | --- | --- |
|  | **Acute**  N = 24 | **72 hours**  N = 52 | **2 weeks**  N = 64 | **3 months**  N = 63 | **12 months**  N = 61 | **Acute**  N = 34 | **72 hours**  N = 97 | **2 weeks**  N = 113 | **3 months**  N = 109 | **12 months**  N = 98 |
| **GFAP (pg/mL)** |  |  |  |  |  |  |  |  |  |  |
| Mean | 836.1 | 432.6 | 65.1 | 46.4 | 43.9 | 495.5 | 215.7 | 49.1 | 41.4 | 40.4 |
| Median | 665.5 | 80.5 | 51.6 | 42.3 | 40.6 | 173.3 | 50.1 | 39.4 | 36.9 | 35.4 |
| SD | 866.4 | 1130.8 | 38.6 | 20.5 | 20.3 | 912.3 | 743.0 | 34.9 | 21.5 | 21.2 |
| IQR | 1043.7 | 220.6 | 45.9 | 22.7 | 22.6 | 351.2 | 78.6 | 24.9 | 18.7 | 16.8 |
| Range | 25.0-3174.7 | 15.1-6815.1 | 12.3-189.8 | 12.0-106.1 | 9.0-94.1 | 37.0-4212.9 | 12.9-5634.3 | 10.2-28.5 | 7.8-138.4 | 5.2-133.9 |
| **NFL (pg/mL)** |  |  |  |  |  |  |  |  |  |  |
| Mean | 8.0 | 9.0 | 45.4 | 13.3 | 5.8 | 6.4 | 6.7 | 23.9 | 10.4 | 6.0 |
| Median | 6.2 | 7.7 | 22.3 | 9.0 | 5.4 | 5.1 | 5.3 | 7.2 | 6.0 | 5.1 |
| SD | 5.6 | 6.4 | 57.4 | 13.0 | 2.4 | 4.1 | 4.6 | 55.6 | 15.4 | 3.8 |
| IQR | 3.8 | 5.4 | 55.2 | 9.4 | 3.0 | 3.6 | 3.7 | 14.5 | 4.9 | 3.4 |
| Range | 3.2-29.0 | 2.1-40.3 | 2.3-235.0 | 2.8-74.5 | 2.2-14.2 | 2.9-25.1 | 1.6-35.5 | 1.6-379.2 | 1.6-88.5 | 2.5-33.7 |
| **Tau (pg/mL)** |  |  |  |  |  |  |  |  |  |  |
| Mean | 4.4 | 2.4 | 2.6 | 2.5 | 2.1 | 3.6 | 2.3 | 2.6 | 2.4 | 2.3 |
| Median | 3.6 | 2.3 | 2.4 | 2.5 | 2.0 | 2.8 | 2.0 | 2.5 | 2.3 | 2.1 |
| SD | 2.5 | 1.1 | 1.2 | 1.1 | 0.9 | 3.0 | 1.5 | 0.9 | 1.2 | 1.2 |
| IQR | 2.3 | 1.0 | 1.1 | 1.3 | 1.1 | 2.0 | 1.3 | 1.0 | 1.3 | 1.3 |
| Range | 1.24-10.2 | 0.7-6.4 | 0.5-8.7 | 0.8-6.1 | 0.2-4.7 | 1.0-17.0 | 0.4-13.5 | 0.8-5.6 | 0.4-6.6 | 0.3-7.1 |

**Supplementary Table 10:** Descriptive statistics of GFAP, NFL and tau concentrations, on their original scale, in patients with mTBI and other somatic injuries (mTBI+) and patients without other somatic injuries (mTBI-), at each timepoint.

mTBI, mild traumatic brain injury; GFAP, Glial fibrillary acidic protein; NFL, Neurofilament light.

|  | **Community Controls** | | | | | **Trauma Controls** | | | | |
| --- | --- | --- | --- | --- | --- | --- | --- | --- | --- | --- |
|  | **Acute**  N = 82 | **72 hours**  N = 0 | **2 weeks**  N = 0 | **3 months**  N = 74 | **12 months**  N = 67 | **Acute**  N = 6 | **72 hours**  N = 5 | **2 weeks**  N = 11 | **3 months**  N = 46 | **12 months**  N = 0 |
| **GFAP (pg/mL)** |  |  |  |  |  |  |  |  |  |  |
| Mean | 38.1 |  |  | 39.3 | 37.6 | 41.1 | 30.1 | 33.1 | 39.9 |  |
| Median | 35.8 |  |  | 38.5 | 37.5 | 33.4 | 32.5 | 26.7 | 37.2 |  |
| SD | 16.9 |  |  | 17.2 | 14.6 | 25.7 | 15.5 | 16.2 | 17.9 |  |
| IQR | 17.1 |  |  | 22.9 | 20.4 | 12.3 | 19.4 | 13.5 | 18.6 |  |
| Range | 6.2-100.0 |  |  | 9.1-96.3 | 14.2-80.2 | 18.5-90.9 | 11.3-50.3 | 13.6-63.5 | 7.0-95.4 |  |
| **NFL (pg/mL)** |  |  |  |  |  |  |  |  |  |  |
| Mean | 5.6 |  |  | 5.5 | 5.8 | 5.2 | 4.5 | 5.4 | 5.0 |  |
| Median | 5.1 |  |  | 5.1 | 5.1 | 5.2 | 3.9 | 4.2 | 4.4 |  |
| SD | 2.7 |  |  | 2.7 | 2.9 | 1.6 | 1.8 | 2.7 | 2.2 |  |
| IQR | 3.4 |  |  | 3.3 | 3.4 | 1.5 | 1.0 | 2.7 | 2.9 |  |
| Range | 1.7-15.6 |  |  | 2.1-17.7 | 1.8-17.3 | 3.0-7.8 | 2.6-7.4 | 3.3-12.4 | 1.5-10.2 |  |
| **Tau (pg/mL)** |  |  |  |  |  |  |  |  |  |  |
| Mean | 2.5 |  |  | 2.5 | 2.4 | 1.8 | 2.0 | 2.0 | 2.5 |  |
| Median | 2.2 |  |  | 2.4 | 2.4 | 1.8 | 2.4 | 2.1 | 2.2 |  |
| SD | 1.3 |  |  | 1.1 | 1.0 | 0.9 | 0.8 | 0.7 | 1.0 |  |
| IQR | 1.4 |  |  | 1.0 | 1.2 | 1.2 | 1.2 | 0.7 | 1.0 |  |
| Range | 0.5-7.4 |  |  | 0.1-6.5 | 0.5-5.0 | 0.9-3.1 | 1.1-2.7 | 0.6-3.1 | 1.0-5.5 |  |

**Supplementary Table 11:** Descriptive statistics of GFAP, NFL and tau concentrations, on their original scale, in community controls and trauma controls, at each timepoint.

mTBI, mild traumatic brain injury; GFAP, Glial fibrillary acidic protein; NFL, Neurofilament light.
